# Lack of FGF21 promotes NASH-HCC transition *via* hepatocyte-TLR4-IL-17A signaling

**DOI:** 10.7150/thno.45988

**Published:** 2020-08-07

**Authors:** Qianqian Zheng, Robert C. Martin, Xiaoju Shi, Harshul Pandit, Youxi Yu, Xingkai Liu, Wei Guo, Min Tan, Ou Bai, Xin Meng, Yan Li

**Affiliations:** 1 Department of Surgery, School of Medicine, University of Louisville, Louisville, KY 40202, USA.; 2 Department of Pathophysiology, Basic Medicine College, China Medical University, Shenyang 110122, China.; 3 Department of Hepatobiliary and Pancreatic Surgery, The First Hospital of Jilin University, Changchun 130021, China.; 4 Department of Hematology, The First Hospital of Jilin University, Changchun 130021, China.; 5 Department of Biochemistry and Molecular Biology, College of Life Science, China Medical University, Shenyang 110122, China.

**Keywords:** Fibroblast growth factor 21, Nonalcoholic steatohepatities, Toll-like receptor 4, IL-17A, Hepatocellular carcinoma

## Abstract

**Rationale:** Hepatocellular carcinoma (HCC) has been increasingly recognized in nonalcoholic steatohepatitis (NASH) patients. Fibroblast growth factor 21 (FGF21) is reported to prevent NASH and delay HCC development. In this study, the effects of FGF21 on NASH progression and NASH-HCC transition and the potential mechanism(s) were investigated.

**Methods:** NASH models and NASH-HCC models were established in FGF21Knockout (KO) mice to evaluate NASH-HCC transition. IL-17A signaling was investigated in the isolated hepatic parenchymal cells, splenocytes, and hepatocyte and HCC cell lines.

**Results:** Lack of FGF21 caused significant up-regulation of the hepatocyte-derived IL-17A via Toll-like receptor 4 (TLR4) and NF-κB signaling. Restoration of FGF21 alleviated the high NAFLD activity score (NAS) and attenuated the TLR4-triggered hepatocyte-IL-17A expression. The HCC nodule number and tumor size were significantly alleviated by treatments of anti-IL-17A antibody.

**Conclusion:** This study revealed a novel anti-inflammatory mechanism of FGF21 via inhibiting the hepatocyte-TLR4-IL-17A signaling in NASH-HCC models. The negative feedback loop on the hepatocyte-TLR4-IL-17A axis could be a potential anti-carcinogenetic mechanism for FGF21 to prevent NASH-HCC transition.

## Introduction

Non-alcoholic fatty liver disease (NAFLD) encompasses a broad spectrum of conditions, ranging from non-progressive bland steatosis to nonalcoholic steatohepatitis (NASH) 1, 2. NASH is the most severe form of NAFLD and a potential precursor of hepatocellular carcinoma (HCC) 3. In previous studies, we found that hepatic fibroblast growth factor (FGF21) protein level increased in steatohepatitis but decreased during HCC development 4. Severe NASH and aberrant pro-inflammatory signaling were also found in the FGF21 knockout (FGF21KO) mice 5. FGF21 is primarily produced in the liver under metabolic stress caused by starvation, hepatosteatosis, obesity and diabetes 6, 7. Under the regulation of peroxisome proliferator-activated receptor α (PPARα) in response to the accumulation of lipids, hepatic FGF21 elicits metabolic benefits, in turn acting on the adipocytes of distal adipose tissue, through the transmembrane receptor FGFR1-coreceptor β-Klotho complex 8. This major endocrine action of FGF21 results in a combination of effects including control of lipolysis, clearance of excessive free fatty acids (FFAs), enhancing expenditure of the stored lipid energy by mitochondrial substrate oxidation, catabolism and uncoupling, and therefore, negatively regulating hepatic or tissue steatosis, and adiposity 9, 10.

Pharmacological application of FGF21 holds great promise as an effective therapeutic means for treating obesity and diabetes 11-13. Using native FGF21, FGF21 analogues or metformin to stimulate FGF21 production, several experimental studies have also implicated the metabolic bioactivities of FGF21 against NAFLD 14-16. In a recent study, FGF21 is found to alleviate inflammation by suppression of T helper 17 (Th17) cell differentiation and IL-17A expression via regulation of adiponectin in a NASH mouse model 17. Other studies have also shown that FGF21 exerts anti-inflammatory efficacy in down-regulation of the Th17-IL-17 axis in experimental models of rheumatoid arthritis 18, 19. In clinical patients, the transition from steatosis to NASH has been demonstrated to be associated with hepatic infiltration of IL-17A-producing cells 20. Accumulating evidence indicated that Th17-IL-17 axis mediates the progression from NASH to HCC 21, 22, while blockage of Th17-IL-17 axis can inhibit this progression 23. All the previous studies build upon the scientific premise that FGF21 is a promising candidate for treating NASH and preventing NASH-HCC transition. However, the mechanism of FGF21, especially its effect on Th17-IL-17 axis during in NASH-HCC transition, has not been well addressed.

Currently, it is speculated that FGF21 may play an anti-inflammatory effect against NASH via inhibition of hepatic Th17 cell infiltration 18, 19, 23. However, the liver is a tolerogenic organ with exquisite mechanisms of immune regulation that ensure upkeep of local and systemic immune tolerance to self and foreign insults 24. Because of immune tolerance, hepatic Th17 cell infiltration and infiltrated cells-derived IL-17A production may be not the common scenario in NASH. As we know, Toll-like receptor 4 (TLR4) serves as an upstream signal of the Th17-IL-17 axis 25. Recent studies have shown that TLR4 mediates inflammation in hepatic parenchymal cells and non-parenchymal cells in the early stages of NAFLD 26, 27, suggesting that the “liver cells” may contribute to the inflammatory events in liver tissue. Revealing the hepatic inflammatory mechanism is critical to help understand the potential anti-inflammatory/anti-cancer action of FGF21.

In this study, a NASH model and a NASH-HCC model were established in FGF21KO mice as well as the wild type controls. Administrations of exogenous rhFGF21 and anti-IL17A antibody in mice were performed to investigate the anti-inflammatory mechanism of FGF21. Benign hepatocyte line and hepatoma cell lines were used to perform *in vitro* studies to explore the hepatocyte-based TLR-4/IL-17A signaling and potential therapeutic targets against NASH-HCC transition. In addition, FGF21 and IL-17A expressions were determined in HCC patients, and analyzed using a web-based database, Gene Expression Profiling Interactive Analysis (GEPIA).

## Results

### Lack of FGF21 worsens the diet-induced NASH in mice

**Figure 1:**
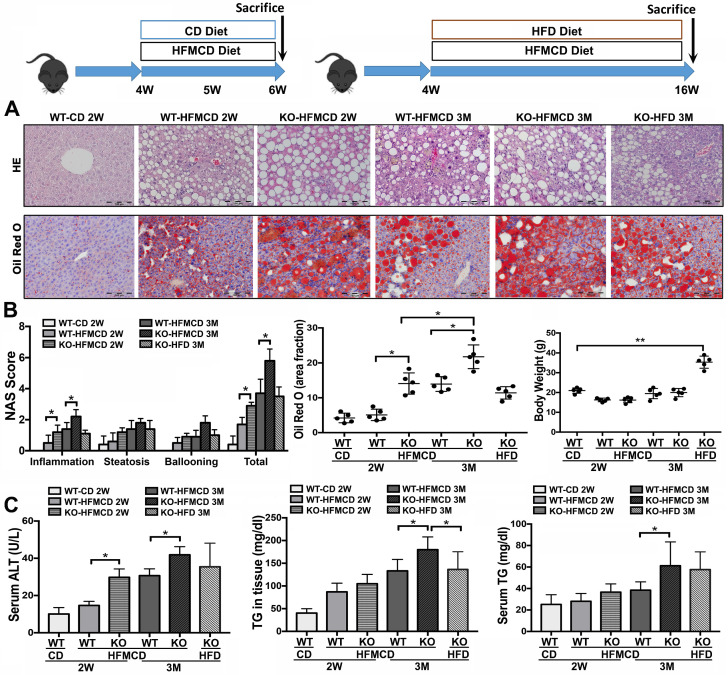
** Establishing NASH models in FGF21KO mice. A.** Representative histology by H&E and Oil Red O staining in the liver tissues from 4 groups (WT-CD; WT-HFMCD 2W; FGF21KO-HFMCD 2W; WT-HFMCD 3M; FGF21KO-HFMCD 3M; and FGF21KO-HFD 3M). For histological details in the H&E staining, bland steatosis was characterized as infiltration of inflammatory cells in the acinar zone and in the form of hepatocyte ballooning being detected. The Oil red O staining confirmed steatosis by identification of the lipid drops. **B.** NAFLD activity score (NAS), computer-imaging analysis for lipid drops of positive Oil Red O staining and Liver weight in all the study groups. NAS was calculated by the sum of scores of steatosis (0-3), lobular inflammation (0-3) and hepatocyte ballooning (0-2). The scoring is conducted as follows: Steatosis: 0, <5%; 1, 5-33%; 2, >33%; 3, > 66. Lobular Inflammation: 0, no foci; 1, <2 foci/200X; 2, 2-4 foci/200X; 3, >4 foci/200X. Hepatocyte Ballooning: 0, no balloon cells; 1, 1-5 balloon cells/200X; 2, >5 balloon cells/200X. **C.** Serum ALT, serum TG levels and hepatic TG levels in all the study groups. KO: FGF21KO; W: week; M: month. *, *P* < 0.05; **, *P* < 0.01.

Based on the previous reports 28-30, methionine-choline deficient (MCD) diet-feeding for 2 weeks and 3 months would be optimal “study-windows” to assess the early and advanced lipid metabolic/inflammatory molecular events. In current study, the FGF21KO mice and WT mice were fed with high-fat MCD (HFMCD) for 2 weeks and 3 months, respectively, while CD and HFD were used as controls. Steatohepatitis was defined in micro-sections with H&E staining, as evident by the pathological changes, and was confirmed by NAS system, which is well-accepted as a surrogate for the histologic diagnosis of NASH 31, 32. Significant increases of serum ALT and TG as well as hepatic TG level were found in the FGF21KO mice with 3 months' HFMCD feeding, compared to all other groups. Consistently, the highest NAS was found in the FGF21KO mice with 3 months' HFMCD feeding, with statistical significance compared to all other groups. The highest NAS was rendered by inflammation score (Figure 1). Significantly up-regulated expressions of proinflammatory cytokines, especially the important components of Th17-IL-17A signaling, were found in FGF21KO-HFMCD mice (Figure S1). The results indicated that lack of FGF21 could decrease the anti-inflammation potential in the liver during NASH development in the HFMCD-fed mice.

### Lack of FGF21 upregulates IL-17A, insulin-resistance and inflammation in NASH mice

**Figure 2:**
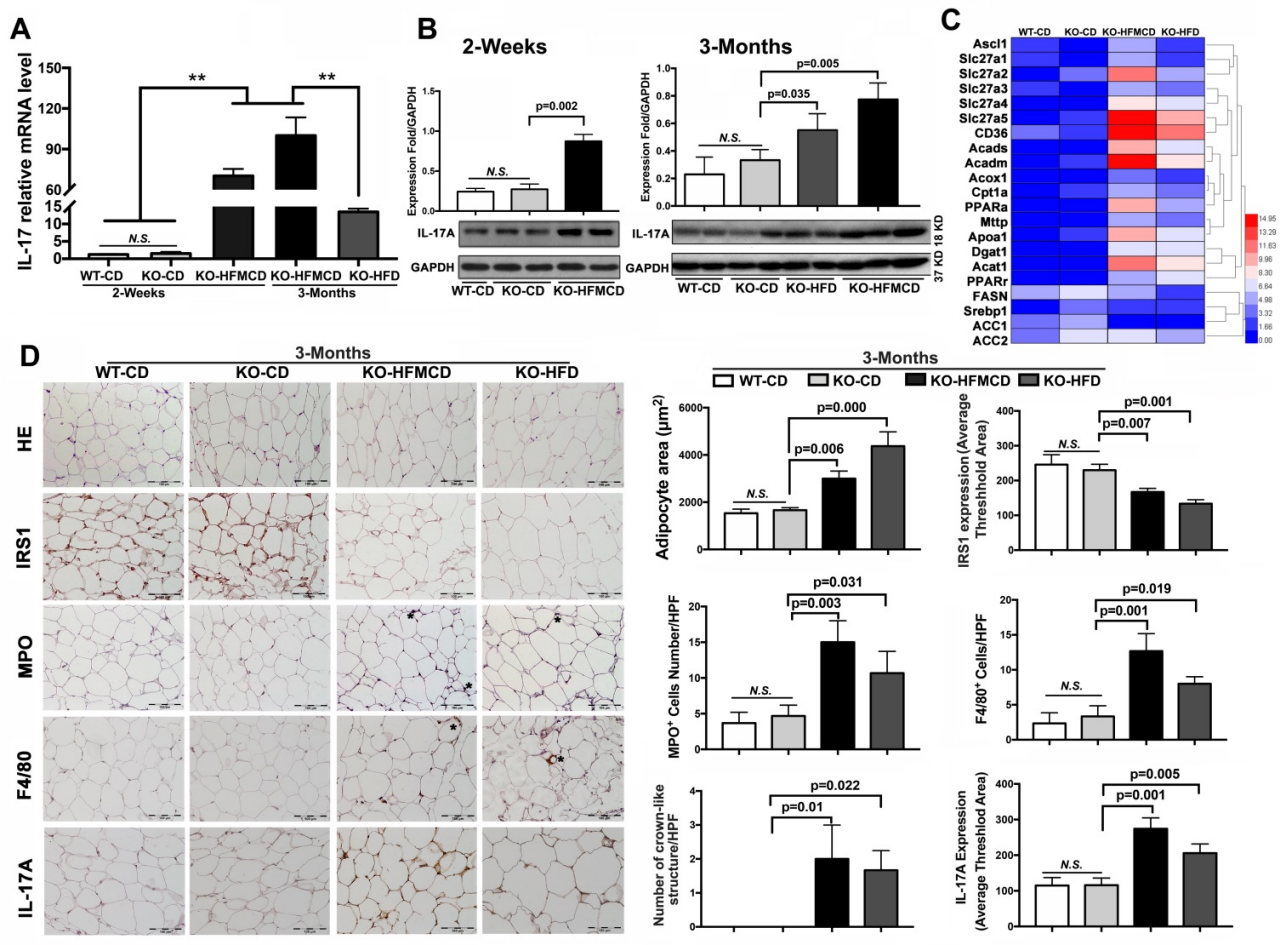
** Aberrant IL-17A and insulin-resistance in NASH mice. A,B.** The expressions of mRNA and protein levels of IL-17A from the liver tissues of FGF21KO mice with HFMCD feeding for 2 weeks and 3 months as well as CD and HFD feeding controls. **C.** Heat map of FFAs metabolic signaling including FA transport (Ascl1, Slc27a1, Slc27a2 Slc27a3, Slc27a4, Slc27a5, and CD36), FA oxidation (Acads, Acadm, Acox1, Cpt1a, and PPARa), export (Mttp and Apoa1), esterification (Dgat1 and Acat1) and FA synthesis (FASN, Srebp1, ACC1, and ACC2) by q-PCR analysis in the FGF21KO mice with HFMCD feeding or HFD feeding for 3 months. **D.** Representative images of histology, IHC for IRS1, MPO, F4/80 and IL-17A, and computer-imaging quantification for the measurement of adipocyte area and the IHC expressions in the WAT from the FGF21KO mice with HFMCD feeding or HFD feeding for 3 months. Positive MPO cells and positive F4/80 cells as well as the number of crown-like structures were counted under microscope at high power field. HPF: high power field; KO: FGF21KO; **, *P* < 0.01.

IL-17A production and IL-17A-producing cells are accepted as key factors contributing to NASH development 33. Considering the etiology and pathology of the Th17-IL-17A axis and its role in steatohepatitis, hepatic infiltration of IL-17A-producing cells was first determined. Subpopulations of CD4^+^/IL-17^+^ cells and CD4^+^/Foxp3^+^ cells were detected by flow cytometry at very low levels in the isolated cells from the liver tissues compared to that in extrahepatic splenocytes (Figure S2-S3). IL-17A expression and production were further determined in liver tissues. IL-17A mRNA expression was significantly upregulated in the FGF21KO HFMCD-fed mice with compared to those with either CD or HFD feeding (Figure 2A). Consistently, IL-17A protein production was significantly increased in the liver tissues of FGF21KO-HFMCD mice (Figure 2B). Up-regulation of fatty acid (FA) transport, FA oxidation, FA esterification and FA synthesis, were also found in the liver tissues of FGF21KO-HFMCD mice (Figure 2C). In NAFLD patients, it is reported that approximately 60% of hepatic lipid accumulation is derived from the re-esterification of plasma free FAs derived from lipolysis because of insulin resistance 34. We further investigated the adipose tissues in terms of insulin-resistance and inflammation. Enlarged adipocytes widely distributed to the white adipose tissue (WAT) but decreased expression of insulin receptor substrate 1 (IRS1) were found in the FGF21KO mice with HFMCD or HFD feeding. Significantly increased neutrophils and macrophages identified by MPO and F4/80 staining and crown-like structures (CLSs), composed of macrophages surrounding dead or dying adipocytes, were found in the FGF21KO mice with HFMCD or HFD feeding. Consistently, significantly increased IL-17A expression was found in adipose tissues of FGF21KO mice with HFMCD or HFD feeding (Figure 2D). The results indicated that lack of FGF21 caused increases of IL-17A production and up-regulation of FA metabolism in liver, and increased the severity of insulin-resistance and inflammation in adipose tissue, in which the increased IL-17A production further induced neutrophil and monocyte recruitment toward inflammation sites 35. Because the liver is considered a tolerogenic organ with the mechanisms for appropriate regulation of immune cells 36, finding resources of IL-17A production in a subpopulation of liver cells is important to understand the NASH mechanism in FGF21KO mice.

### Lack of FGF21 up-regulated IL-17A production of hepatic parenchymal cells

**Figure 3:**
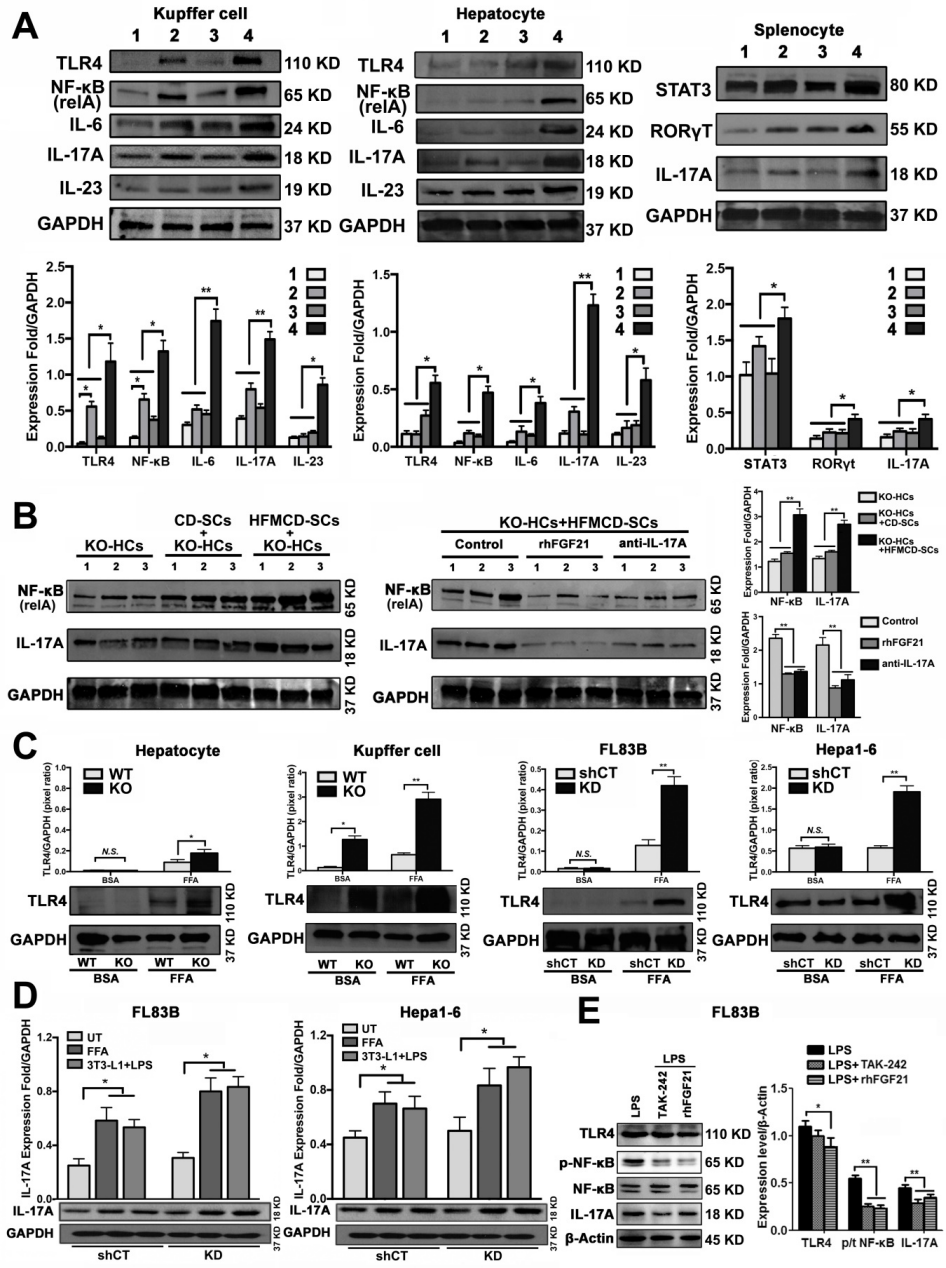
** Up-regulated IL-17A production of hepatic parenchymal cells. A.** Western blot analysis for the protein levels of TLR4, NF-κB (p65, relA), IL-17, IL-6, and IL-23 in the isolated Kupffer cells and hepatocytes as well as for the protein levels of STAT3, RORγT, IL-17 in the isolated splenocytes from early stage study groups (2 weeks) (1: WT-CD; 2: WT-HFMCD; 3: FGF21KO-CD; 4: FGF21KO-HFMCD). **B.** Western blot analysis for the protein levels of NF-κB and IL-17A in the FGF21KO-hepatocytes co-cultured with the splenocytes isolated from the spleens of FGF21KO-CD mice and FGF21KO-HFMCD mice. Monoclonal anti-mouse IL-17A antibody and rhFGF21 were used to treat the co-cultured cells at 100 ng/mL for 24 h. **C.** Western blot analysis for the protein levels of TLR4 in the primarily cultured hepatocytes and Kupffer cells (from the mice both FGF21KO and WT mice) as well as the hepatic cell lines (FL83B cells, Hepal-6 cells, FL83B-FGF21KD cells and Hepal-6-FGF21KD cells). The cells were challenged with FFA (palmic acid) at 100uM for 48 h, 1% BSA was used as treatment control. **D.** Western blot analysis for the protein levels of IL-17A in the hepatic cell lines (FL83B cells and Hepal-6 cells) and the associated cells of FGF21 gene knockdown by shRNA, named FL83B-FGF21KD cell and Hepal-6-FGF21KD cell, which were co-cultured with adipocyte line, 3T3-L1 cells. **E.** Western blot analysis for the protein levels of TLR4, phosphorylated (p)-NF-κB, NF-κB (p65, relA), and IL-17A in FL83B cells challenged with LPS and treated with TAK-242 and rhFGF21. HCs: hepatocytes; SCs: splenocytes; KO: FGF21KO; KD: FGF21KD; FFA: free fatty acid; shCT: shControl.* N.S*.: no statistical significance; *, *P* < 0.05; **, *P* < 0.01.

**Figure 4:**
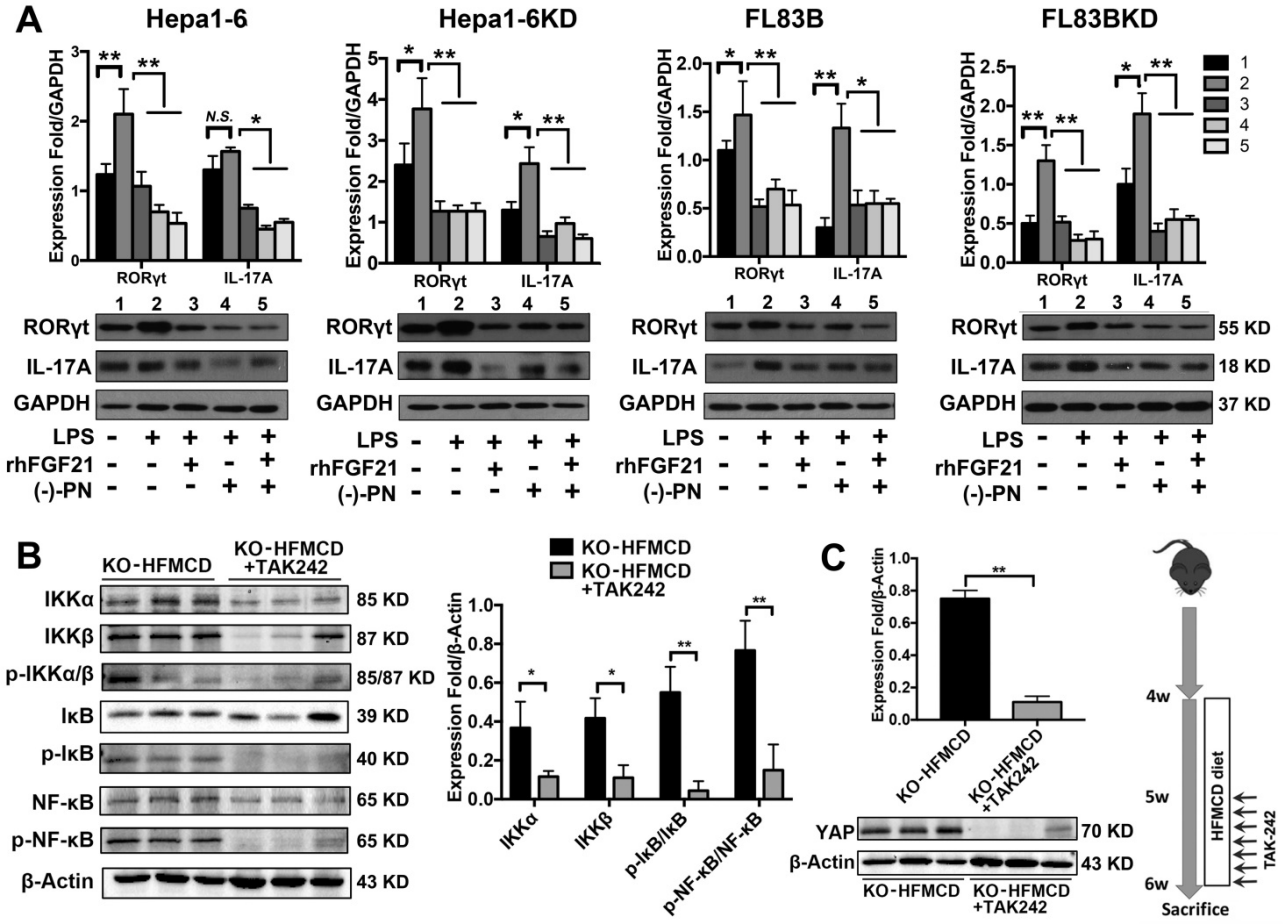
** TLR4-NF-κB signaling contributed to IL-17A production and attenuation of IL-17A and YAP via inhibition of TLR4 in hepatocytes. A.** Western blot analysis for the protein levels of RORγt and IL-17A in the FL83B/FL83B-FGF21KD cells and Hepal-6/Hepal-6-FGF21KD cells challenged with LPS and treated with (-)-parthenolide or rhFGF21. **B.** Western blot analysis for the protein levels IKKα, IKKβ, p-IκB/IκB, and p-NF-κB/NF-κB in the hepatocytes isolated from the FGF21KO-HFMCD mice and FGF21KO-HFMCD+TAK-242. **C.** Western blot analysis for the protein level of YAP in the hepatocytes isolated from the FGF21KO-HFMCD mice and FGF21KO-HFMCD+TAK-242. KO: FGF21KO; KD: FGF21 KD; FFA: free fatty acid; SIM: simvastatin; (-)-PN: (-)-parthenolide; *N.S*.: no statistical significance; *, *P* < 0.05; **, *P* < 0.01.

Hepatocytes, Kupffer cells and extrahepatic splenocytes were isolated from liver and spleen to determine IL-17A production and IL-17A signaling. It was interesting to find that significantly increased IL-17A production was detected in hepatocytes, in addition to Kupffer cells and splenocytes from FGF21KO-HFMCD mice. The initiating cytokines, IL-23 and IL-6, for Th17 lineage 37 and the upstream signals (STAT3, RORγt, NF-κB and TLR4) for IL-17A were determined in the isolated subpopulations. Significantly up-regulated expressions of IL-23, IL-6, NF-κB, and TLR4 were found in Kupffer cells and hepatocytes of FGF21KO-HFMCD mice, while up-regulated transcription factors of both STAT3 and RORγt for Th17 lineage were detected in the splenocytes of FGF21KO-HFMCD mice (Figure 3A). This result indicated that the IL-17A could be produced in FGF21KO-hepatocytes via cross-talk with extrahepatic splenocytes. To confirm, an indirect co-culture assay was performed using the isolated hepatocytes and splenocytes. Significantly up-regulated protein levels of IL-17A and NF-κB were found in the FGF21KO-hepatocytes when co-cultured with splenocytes from FGF21KO-HFMCD mice, while treatment with either rhFGF21 or anti-IL-17 antibody significantly attenuated the up-regulated protein levels of NF-κB and IL-17A (Figure 3B). TLR4-mediated inflammation played an important role in liver diseases 26, 27. However, most previous studies emphasized the pathophysiological importance of TLR4 from non-parenchymal cells such as Kupffer cells/macrophages 38, 39 and hepatic stellate cells 40. The aberrant TLR4 signaling from hepatocytes, which make up approximately two-thirds of total liver cell population (60%-70%), could be more deleterious but there was no such study previously. We further investigated TRL4 expression in the primary culture of hepatocytes from FGF21KO mice. Unlike the Kupffer cells, very low levels of TRL4 expression were detected in the hepatocytes from FGF21KO mice as well as WT mice. However, when the cells were challenged by FFA, a moderate increase of TLR4 was found in WT-hepatocytes, but a significant increase of TLR4 was found in FGF21KO-hepatocytes. TLR4 expression was also studied in a benign cell line of hepatocyte (FL83B cells) and an HCC cell line (Hepal-6 cells) using a shRNA assay to knock down (KD) the FGF21 gene. Similarly, FFA challenging caused significant increases of TLR4 in either FL83B-FGF21KD cells or Hepal-6-FGF21KD cells (Figure 3C). The results indicated that lack of FGF21 could leave the hepatocytes to become an important resource of IL-17A production, while FFA played an important role in contributing to hepatocyte-IL-17A production. On the other hand, IL-17A protein also worsened TLR4/NF-κB/IL-17A signaling and lipid accumulation in hepatocytes, especially in the FGF21KD cells (Figure S4-S5). Because adipose-derived FFA was a major resource contributing to steatosis in NASH, a co-culture assay was performed to co-culture adipocytes with hepatocytes. Similar to FFA challenging, co-culture with LPS stimulated 3T3-L1 cells induced significant increases of IL-17A production in FL83B-FGF21KD, Hepal-6 and Hepal-6-FGF21KD cells (Figure 3D), which implies that LPS-induced FFA release contributed IL-17A production in hepatocytes. To explore the regulatory mechanism of FGF21 on hepatocyte-TLR4 signaling, FL83B cells were challenged with LPS and treated with TAK242, an inhibitor of TLR4, and rhFGF21 to investigate TLR4 and the down-stream signals including NF-κB and IL-17A. The results indicated that the LPS-induced increases of NF-κB and IL-17A were attenuated by either TAK242 or rhFGF21 (Figure 3E). To confirm the participation of TLR4-NF-κB signaling in IL-17A production, NF-κB signaling was inhibited by (-)-parthenolide, an inhibitor of NF-κB, to investigate IL-17A as well as RORγt, a master transcription factor of IL-17 expression, in either FL83B/FL83B-FGF21KD cells or Hepal-6/Hepal-6-FGF21KD cells. The results showed that treatments with either (-)-parthenolide or rhFGF21 attenuated significantly the expressions of IL-17A and RORγt (Figure 4A). An *in vivo* study was also performed in FGF21OK mice treated with TAK242 to confirm the participation of TLR4-NF-κB signaling in early NASH model (2 weeks). The results indicated that inhibition of TLR4 attenuated significantly the expressions of IKKα, IKKβ, p-IκB/IκB, and p-NF-κB/NF-κB in the isolated hepatocytes from liver tissues of NASH model (Figure 4B). Interestingly, TAK242 treatment also attenuated significantly the expression of YAP (Figure 4C), an important component of HIPPO pathway. All the data evoked the interest to further study whether FGF21 could protect hepatocytes via negative feedback to the hepatocyte-derived IL-17A production, and thereby prevent NASH-HCC.

### rhFGF21 prevents NAHS via inhibition of the TRL4 mediated IL-17A production

**Figure 5:**
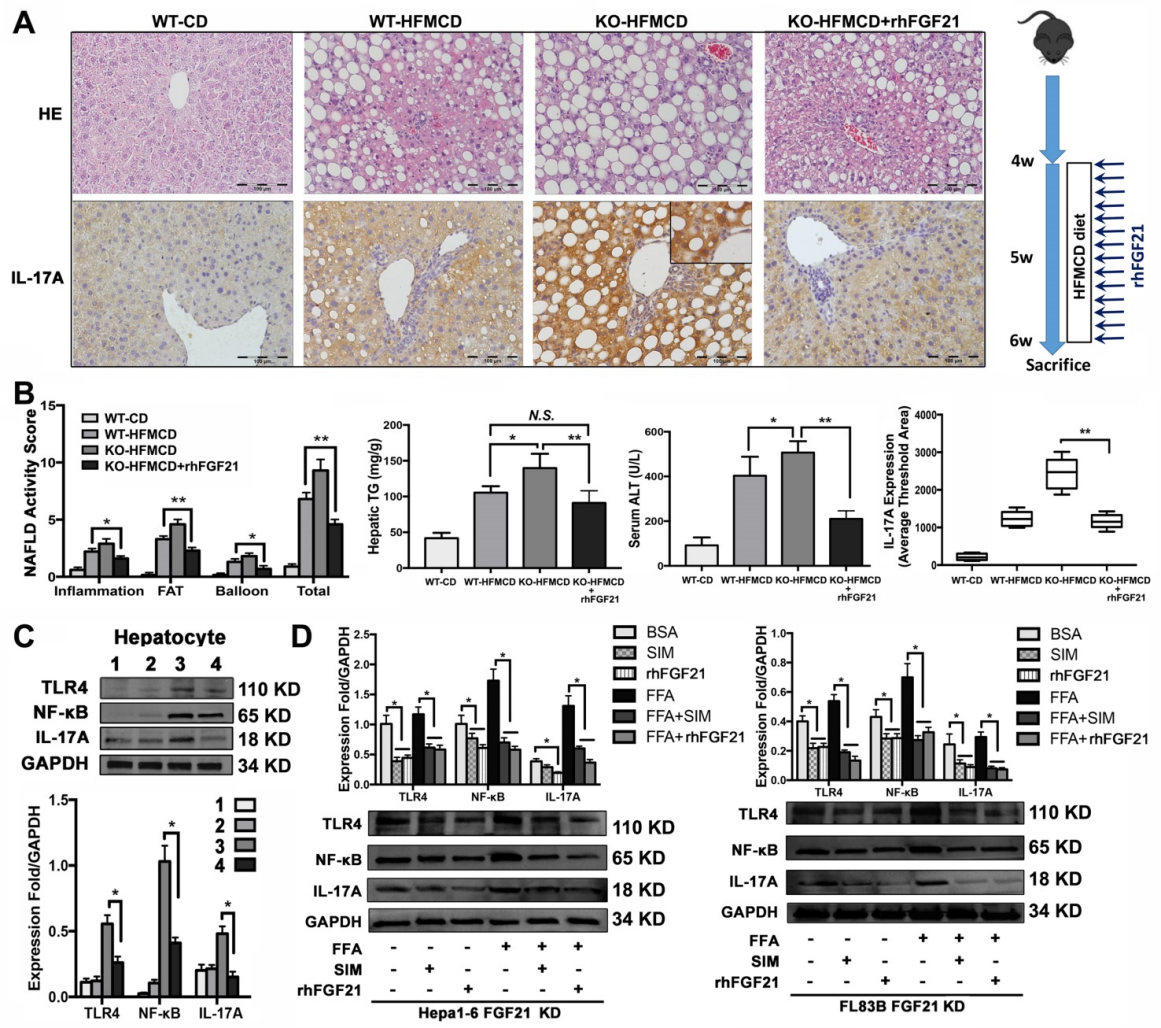
** rhFGF21 preventing NAHS via inhibition of IL-17A production. A.** Representative histology by H&E stain and IL-17 expression by IHC staining in the liver tissues from 4 groups (WT-CD; WT-HFMCD; FGF21KO-HFMCD; FGF21KO-HFMCD+rhFGF21). **B.** The NAFLD activity score (NAS), hepatic TG, serum ALT and computer-imaging quantification of IL-17 expression were determined in all 4 groups. NAS was calculated as aforementioned. **C.** Western blot analysis for the protein levels of TLR4, NF-κB, and IL-17A in the hepatocytes isolated from the 4 group mice (1: WT-CD; 2: WT-HFMCD; 3: FGF21KO-HFMCD; 4: FGF21KO-HFMCD+rhFGF21). **D.** Western blot analysis for the protein levels of TLR4, NF-κB and IL-17A in the FL83B-FGF21KD cells and Hepal-6-FGF21KD cells treated with FFA, SIM and rhFGF21, respectively. KO: FGF21KO; KD: FGF21 KD; FFA: free fatty acid; SIM: simvastatin; *N.S*.: no statistical significance; *, *P* < 0.05; **, *P* < 0.01.

In the early NASH model, alleviation of NASH pathology characterized by decreases of lipid accumulation and inflammatory infiltration in the liver tissues of mice with rhFGF21 treatment. Consistently, the NAS, hepatic TG and ALT were significantly decreased in mice with rhFGF21 treatment. Computer-imaging analysis in IHC showed significant decreases of IL-17A levels in liver tissues with rhFGF21 treatment (*P* < 0.01) (Figure 5A, B). Significantly down-regulated expressions of proinflammatory cytokines and IL-17A signaling and decreased Th17 cell/Treg cell ratio were found in FGF21KO-HFMCD mice with rhFGF21 treatment (Figure S6). The results indicated that restoration of FGF21 was critical in protecting the liver from the HFMCD insults in FGF21KO mice, while a decrease IL-17A might play a key role in hepatic protection. Having determined the hepatic protection by rhFGF21, we next explored if restoration of FGF21 could modulate TLR4/NF-κB/IL-17A signaling in hepatocytes. In the isolated hepatocytes from 4 study groups, rhFGF21 treatment significantly attenuated the up-regulated protein levels of TLR4, NF-κB and IL-17A in the FGF21KO-HFMCD mice (Figure 5C). To further elucidate the effect of FGF21 on hepatocyte-TLR4 signaling, simvastatin (SIM), an inhibitor of the TLR4 signal, was used to treat the FL83B-FGF21KD and the Hepal-6-FGF21KD cells that were challenged with FFA. The results indicated that rhFGF21 was similar to SIM in attenuating the up-regulated expressions of TLR4, NF-κB and IL-17A (Figure 5D). Taken together, Inhibition of hepatocyte-IL-17A production could be an important mechanism for FGF21 to prevent NASH via a negative feedback loop on TLR4/NF-κB/IL-17A signaling.

### Anti-IL-17A inhibits HCC progression in FGF21KO mice

**Figure 6:**
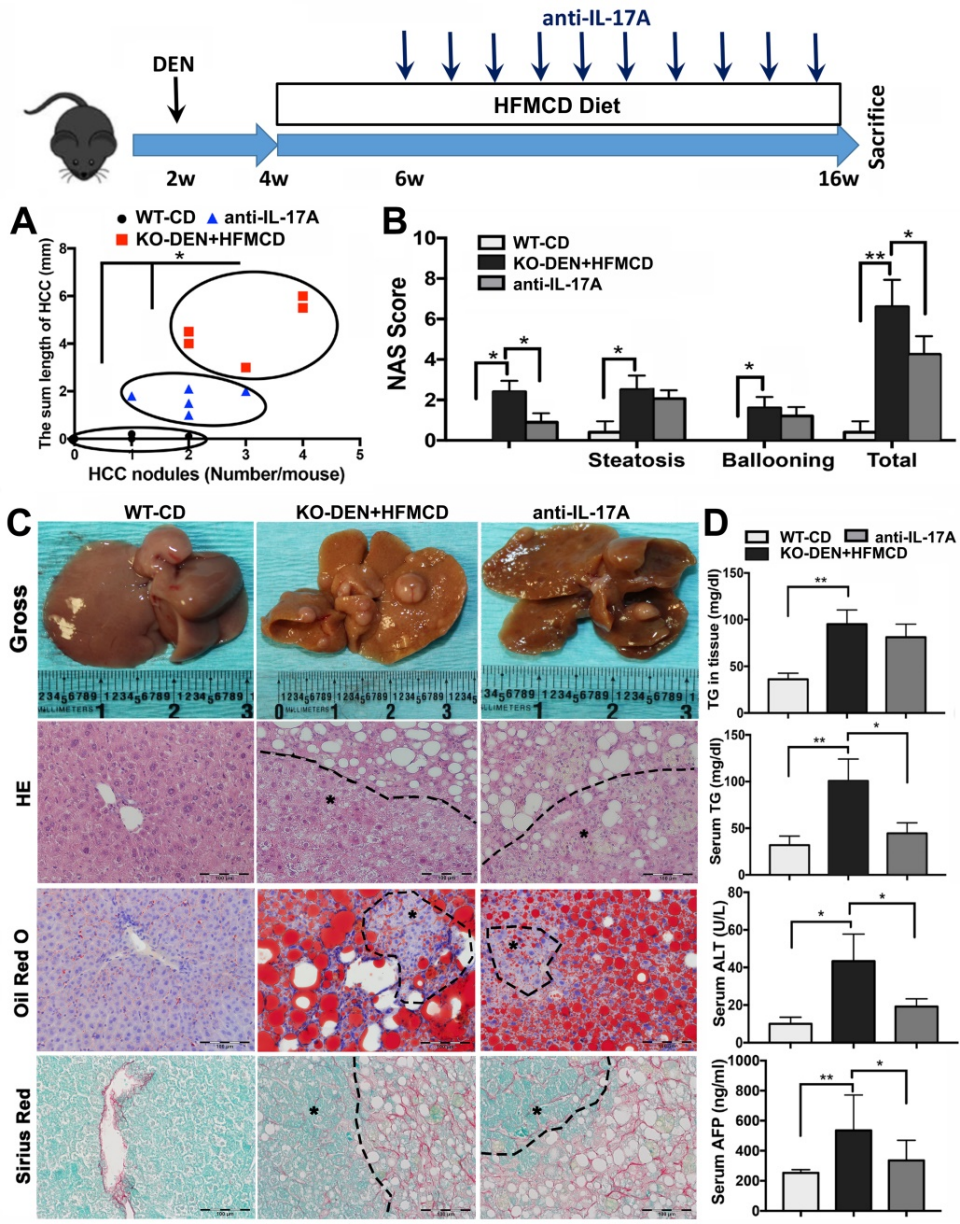
** Anti-IL-17A inhibits NASH-HCC progression in FGF21KO mice. A.** The NASH-HCC tumor growth pattern represented as the regression of HCC nodule length and HCC nodule numbers in individual HCC mouse. **B.** The NAFLD activity score (NAS). **C.** Representative gross anatomy of HCC nodules and histology by staining of H&E Oil Red O and Sirius Red in NASH-HCC mice with treatment of anti-IL-17A antibody. B: Hepatic TG levels, serum TG levels, serum ALT and serum AFP in the study groups. KO: FGF21KO; *, *P* < 0.05; **, *P* < 0.01.

Because IL-17A signaling was accepted as a crucial link for the transition from NAFLD to HCC 41, we further performed an anti-IL-17A treatment in a NASH-HCC transition model established by DEN injection followed by HFMCD/HFD feeding in FGF21KO mice (Figure S7). By ultrasound imaging, tumor nodule was detected at 6 weeks after DEN (2 weeks after HFMCD), while multiple and bigger nodules were detected at 16 weeks after DEN (12 weeks after HFMCD). No tumor nodule was detected in DEN+HFD mice. Based on the results of DEN+HFMCD versus DEN+HFD, the DEN+HFMCD model was selected and an anti-IL-17A treatment window was determined from 6 weeks to 16 weeks to assess its effect on NASH-HCC transition and HCC progression. All FGF21KO mice with DEN+HFMCD developed HCC at 16 weeks, however, lower burden of liver tumors was found in the mice with anti-IL-17A treatment. Macroscopically, smaller size and fewer numbers of HCC nodules were found in the mice with anti-IL-17A treatment compared to the untreated mice. When a scatter plot was created with length and number of the HCC nodules to present tumor growth patterns, significantly alleviated tumor growth was found in anti-IL-17A treated mice compared to the untreated mice (Figure 6A). Decreased NAS was also found in the anti-IL-17A treated mice, with statistical significance compared to the untreated mice. The NAS result showed that the decreased total NAS was from the decreased score of inflammation other than steatosis and ballooning (Figure 6B), supporting the anti-inflammatory effect of anti-IL-17A. The HCC nodules were confirmed as HCC foci by the cytological features of cancerous cells; ranging from well to poorly differentiated; distributed in parenchyma showing an abnormal hepatic architecture. NASH pathology was further evidenced by lipid accumulation using Oil Red O staining and by fibrosis using Sirius Red staining (Figure 6C). Significant decreases of serum TG, ALT and AFP were found in the anti-IL-17A treated mice compared to the untreated mice (Figure 6D). Taken together, lack of FGF21 accelerates the NASH-HCC transition via up-regulation if IL-17A signaling. FGF21 could be important in the protection of hepatocytes against NASH-HCC transition via a negative feedback loop on IL-17A signaling in hepatocytes.

### Aberrant signaling of FGF21/ IL-17A /YAP in human HCC

**Figure 7:**
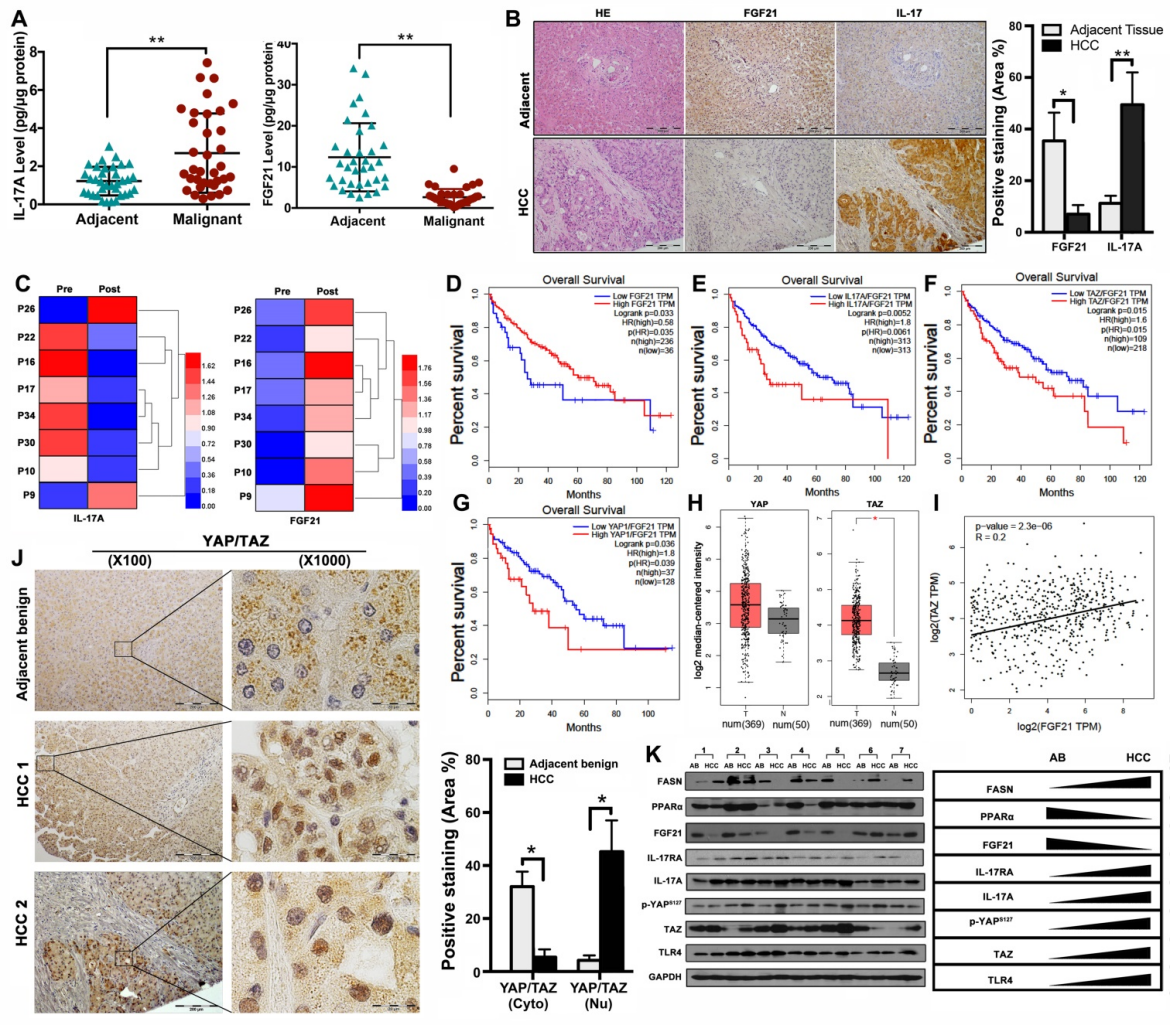
** Aberrant signaling of FGF21/ IL-17A /YAP in human HCC. A.** Protein levels of IL-17A were analyzed by ELISA assay in HCC patients' malignant tissues as well as adjacent benign tissues. **B.** Representative histology by H&E staining and computer-imaging quantification of FGF21 and IL-17A expressions by IHC staining in patients' HCC tissues as well as adjacent benign tissues. **C.** Protein levels of IL-17A were analyzed by ELISA assay in patients' HCC pre-operation and post-operation. **D-G.** FGF21 mRNA expression levels and the FGF21 normalized YAP/TAZ/IL-17A mRNA expression levels in HCC patients associating with clinical outcomes. Low mRNA expression of FGF21 was correlation with poor prognosis in LIHC. High mRNA expression of IL-17A was correlation with poor prognosis in LIHC, as well as YAP and TAZ. Red, high expression; blue, low expression. **H.** log2 median-centered intensity of YAP expression and TAZ expression in the HCC patients (T) and Non-HCC patients (N). The GEPIA database revealed that TAZ expression was significantly upregulated in HCC. The boxplot analysis show log2 (TPM + 1) on a log-scale. T=369, N=50. **I.** The correlation between the mRNA expression levels of TAZ and FGF21 was determined in the TCGA-LIHC dataset analyzed using GEPIA. **J.** Representative images of YAP/TAZ distribution in cytosol and nuclear by IHC staining and computer-imaging quantification YAP/TAZ expressions in patients' HCC tissues as well as adjacent benign tissues. **K.** Western blot analysis for the protein levels of FASN, PPARα, FGF21, IL-17A, IL-17RA, p-YAP^s127^, TAZ, and TLR4 in patients' HCC tissues as well as adjacent benign tissues. AB: adjacent benign. *, *P* < 0.05; **, *P* < 0.01.

The data from NASH-HCC mice suggested that the FGF21-IL-17A axis could play an important role during the NASH-HCC carcinogenetic process. Further study was performed in human HCC specimens. The protein levels of FGF21 and IL-17A were determined by ELISA assay in the HCC tissues as well as the adjacent benign tissues. Significantly increased IL-17A was found in the HCC tissues compared to the adjacent benign tissues. In contrast, significantly decreased FGF21 was found in the HCC tissues compared to the adjacent benign tissues (Figure 7A). The IHC analysis was confirmed by the ELISA results (Figure 7B). We also determined the serum FGF21 and IL-17A by ELISA assay in the 8 HCC patients before and after HCC-nodule resection. The results indicated that the aberrant IL-17A levels were alleviated in 6 out of 8 patients, while the FGF21 levels were increased in all patients post-operatively (Figure 7C). To further identify the clinical relevance of FGF21 in human HCC, the survival rate of HCC patients associated with FGF21 expression was determined using a web-based database, Gene Expression Profiling Interactive Analysis (GEPIA). The results indicated that the high expression of FGF21 was positively correlated with a better prognosis in HCC patients (Figure 7D). IL-17A expression and carcinogenetic pathways in the GEPIA were further analyzed to explore the potential carcinogenetic signaling linked to the FGF21 and survival. When normalized by FGF21, survival curves showed a significant negative correlation with the mRNA levels of IL-17A, YAP and TAZ (both YAP and TAZ are the main components in the Hippo pathway) (Figure 7E-G). The mRNA levels of YAP and TAZ were further analyzed in HCC and non-tumor liver tissues using the GEPIA web tool. The results indicated that the YAP and TAZ mRNA levels were increased in LIHC (Liver hepatocellular carcinoma, n=369) compared to the normal sample (n = 50), while TAZ showed statistical significance but not YAP (Figure 7H). By co-expression analysis, there was a positive correlation between TAZ and FGF21 expression in LIHC (R = 0.2,* P* < 0.001) (Figure 7I). To confirm the data from GEPIA, IHC was performed using an anti-YAP/TAZ antibody in the HCC tissues as well as in adjacent benign tissues. The positive staining of YAP/TAZ was abundantly distributed in the nuclei of tumor cells in HCC tissues, however, abundantly distribution of YAP/TAZ was found in the cytosol of hepatocytes in adjacent benign tissues. Image-analysis showed significantly increased YAP/TAZ in nuclei, compared to the adjacent benign tissues (Figure 7J). Consequently, the important components (FASN, PPARα, FGF21, IL-17A, IL-17RA, p-YAP TAZ and TLR4) related to the HCC carcinogenetic transformation were further evaluated by Western blot in the paired human samplers (malignant versus benign) of HCC patients. The results showed that upregulated protein levels (FASN, IL-17A, IL-17RA, p-YAP TAZ and TLR4) and downregulated protein levels (PPARα and FGF21) were found in malignant tissues compared to the adjacent benign tissues (Figure 7K).

## Discussion

In this study, we reported, for first time, that FGF21 plays a critical role for negative feedback on the hepatocyte-derived IL-17A production to attenuate NASH development and NASH-HCC transition. The major signaling components of the upstream and downstream signals of FGF21-IL-17A loop and the potential working hypothesis of FGF21 on NASH and NASH-HCC transition are shown as a schematic diagram in Graphical Abstract.

FGF21 is predominantly expressed in hepatocytes and can be induced under hepatic stress 42. Sufficient FGF21 not only directly reduces hepatic lipid accumulation in an insulin-independent manner 43, 44, but also inhibits the lipolysis of WAT and further decreases circulating FFAs levels 45. All these metabolic bioactivities of FGF21 were considered to protect liver from the insults of the aberrant lipid accumulation. However, the metabolic benefits only were not enough to explain the therapeutic effect of FGF21 on NASH, which involved more progressive cellular events including inflammation, cell death and fibrosis progresses 46. The liver is constantly exposed to toxic and microbial products, but no obvious damage occurs because a healthy liver can regulate inflammation and innate immune responses through protective signaling, such as the TLR signals; this protection is known as “liver tolerance”^24^. Breakdown of the liver tolerance may induce an inappropriate immune response, resulting in acute or chronic inflammatory liver diseases.

TLR4-mediated inflammation in liver cells serves an important role in NAFLD, however most previous studies focused on the TLR4 signaling in nonparenchymal cells such as Kupffer cells and hepatic stellate cells 26, 27. Constituting over 60% of liver cells, hepatocytes are the principal site for the innate immune system to recognize the pathogen-associated molecules via pattern recognition receptors 47. In our study, up-regulated TLR4 expression was found in the FGF21KO-hepatocytes, especially when the FGF21KO hepatocytes challenged by FFAs, suggesting a negative feedback role of FGF21 on the TLR4 signal in hepatocytes. While the hepatocyte-TLR4 is expressed at a very low levels and weakly responds to LPS and other inflammatory mediators 47, 48, the up-regulated TLR4 expression in FGF21KO-hepatocytes may be mediated by FFAs, which is supported by the followings: 1) Accumulation of FFAs in the liver causes hepatocyte injury by the intracellular FFA intermediates, such as diglycerides and ceramides, which are accepted as lipotoxic compounds via activation of TLR4^49^; 2) Because a major function of FGF21 is to prevent lipolysis 10, lack of FGF21 accelerates lipolysis to release FFAs which are accumulated in liver. Although the hepatocyte expressed TLR4 was not widely reported, the mRNA expression of hepatocyte-TLR4 was detected in the primary activated cultured hepatocytes and the hepatocyte-TLR4 was activated in responds to TLR4 ligands 50. In addition, very low levels of TLR4 expression in hepatocytes have been reported in an *in vivo* study 51, which is consistent with our results detecting a low level of TLR4 in hepatocytes from WT mice. In FGF21KO-hepatocytes, however, a higher level of TLR4 expression was found and further up-regulated in response to FFA challenging. rhFGF21 treatment attenuated the FFA-mediated TLR4-IL-17A signaling, rendering FGF21 an anti-inflammatory ability in addition to its metabolic activity. All these data indicated that FGF21 was indispensable for modulation of TLR4-IL-17A signaling to maintain “liver tolerance”.

Hepatocyte death 52 and activation of TLR4 49 by lipotoxic intermediates are critical to initiate immune response and inflammation. It is considered that innate immune cells locate in non-lymphoid tissues where they are poised to respond immediately to tissue injury or pathogenic insults. Our results agreed with the general concept that Th17 cells could develop from naïve CD4^+^ T cell precursors via cytokines to initiate cross-talk between liver cells and innate immune cells 53 in NASH, while a lack of FGF21 could accelerate this process. However, the FFA-mediated hepatocyte-TLR4 activation could be deleterious in clinical NASH patients but it had not been noted previously. The most important finding in the current study was the increased IL-17A via NF-κB in the hepatic parenchymal hepatocytes from FGF21KO mice with HFMCD feeding. Comprising two-thirds of total liver cell population, hepatocytes could be an important resource contributing to IL-17A production, which was considered as a crucial link for NASH-HCC transition 41. In our NASH-HCC model, an aggressive growth pattern of tumor nodules was detected in the FGF21KO mice with a single injection of DEN and HFMCD feeding, while HCC lesions, in terms of nodule number and nodule size, was significantly alleviated when the animals were treated with anti-IL-17A antibody. All the data indicated that lack of FGF21 aggravated the TLR4-mediated up-regulation of IL-17A in hepatocytes, contributing to NASH-HCC transition. There could be a potential anti-carcinogenetic mechanism for FGF21 via a negative feedback loop on the TLR4-IL-17A axis in the liver; this hypothesis was supported by the findings in which high expression of FGF21 and low expression of IL-17A were closely correlated with better prognosis in the HCC patients. Based on the data from GEPIA and our HCC samples from clinical patients, the major components (YAP and TAZ) in HIPPO pathway were closely associated with the FGF21 and IL-17A expression levels in regard to the prognosis. Accumulating evidence suggests that HIPPO pathway plays a critical role linking the NASH-HCC transition 54-56. Further study is needed to address the accurate role of YAP and TAZ, which could be important carcinogenetic signaling involved in the NASH-HCC initiation and progression.

In conclusion, lack of FGF21 contributed to FFA-mediated induction of hepatocyte-TLR4 signaling to up-regulate IL-17A expression in hepatocytes. The potential anti-inflammatory effect of FGF21 could be through a negative feedback on the hepatocyte-TLR4 signaling to inhibit IL-17A production in the liver. The negative feedback loop on the hepatocyte-TLR4 -IL-17A axis could be a potential anti-carcinogenetic mechanism for FGF21 to prevent NASH-HCC transition. This finding highlighted the potential pharmacological application of FGF21, especially for its anti-inflammatory effect, as a promising therapeutic strategy for clinical application to treat NASH at an early stage and thereby prevent NASH-HCC transition.

## Materials and Methods

### Establish NASH and NASH-HCC models

Male FGF21 Knockout (FGF21KO) mice with C57 BL/6J background were generously granted by Dr. Steve Kliewer (University of Texas Southwestern Medical Center). Wild-type (WT) C57 BL/6J mice were obtained from Jackson Laboratory (Bar Harbor, ME). For the NASH model, 4-week-old male mice were fed with HFMCD (L-amino acid diet with 60 kcal% fat, 0.1% methionine and no added choline, A06071302, Research Diets, Inc., New Brunswick, NJ) for 2 weeks (early stage) or 3 months (advance stage), according to previous report^29^, ^30^. For the NASH-HCC model, male littermates, yielding the F1 generation, at 15 days of age received N-nitrosodiethylamine (DEN) (Sigma, St. Louis, MO) at 40 mg/kg by intraperitoneal injection (i.p.) according to our previous study 57. When the mice with DEN administration were 4 weeks old, they were fed HFMCD diet for 3 months. Control diet (CD, 10% kcal% fat, D12450B, Research Diets, Inc., New Brunswick, NJ) and high fat diet (HFD) (Rodent Diet with 60% kcal% fat, D12492, Research Diets, Inc., New Brunswick, NJ) were used. Treatments in animals were as follows: Recombinant human FGF21 (rhFGF21) (#100-42; PeproTech; Rocky Hill, NJ) was injected subcutaneously every day at 125 µg/kg bodyweight for 2 weeks; InVivoMAb anti-mouse IL-17A (#BE0173, clone 17F3, Bio X Cell, Lebanon, NH 03766 USA) was injected intraperitoneally at 10 μg/kg bodyweight twice a week for 10 weeks. TAK-242 (Resatorvid, #614316, Millipore Sigma, MA), was injected intraperitoneally every day at 3 mg/kg bodyweight for 1 week. Six to eight mice were assigned in each group. The animal procedures were approved by the Institutional Animal Care and Use Committee of the University of Louisville, which is certified by the American Association for Accreditation of Laboratory Animal Care.

### Biochemical analysis, histology and NAFLD activity score

Serum alanine aminotransferase (ALT) was measured using an ALT Infinity Enzymatic Assay Kit (ThermoFisher Scientific Inc., Waltham, MA). Triglyceride (TG) was determined using a mouse Triglyceride Colorimetric Assay Kit (Cayman Chemical Company, CA). For histology, the harvested liver tissues were fixed in 10% buffered formalin for 2 weeks and then dehydration was done through graded alcohol series. The dehydrated tissues were cleared using xylene, embedded in paraffin, and sectioned at 4-5 μm slices. Hematoxylin and eosin (H&E) staining was performed in the micro-section to investigate the histopathological damage in the liver from all study groups. The images were reviewed and analyzed under microscope (Olympus 1X51, Olympus Corporation, Tokyo) at 20X magnification. The NAFLD activity score (NAS) was calculated from the sum of the individual scores for steatosis, inflammation and ballooning.

### Cell isolation, cell culture, co-culture and treatments

Isolation of hepatocytes, Kupffer cells and splenocytes from liver and spleen tissues, establishment of Hepa1-6-FGF21KD cell and FL83B-FGF21KD cell, and method for 3T3-L1 cell differentiation are described in supplemental file. An indirect co-culture assay was performed for the isolated hepatocytes and splenocytes. In brief, the isolated hepatocytes were seeded at 1 × 10^5^ in 24-well plate and cultured for 24 h in DMEM supplemented with 10% FBS and penicillin/streptomycin. The isolated splenocytes were pretreated with concanavalin A at 5 µg/mL to stimulate IL-17A and seeded at 1 × 10^4^ in the insert of Transwell cell culture plates (Corning Incorporated, Corning, NY, USA). Then hepatocytes and splenocytes were co-cultured, without refreshing the medium, for 72 h. For FFA challenging, palmic acid (PA) (Sigma-Aldrich, USA) at 100 uM was used to treat the cells, and 1% BSA was used as treatment control. To determine the effects of FGF21 and IL-17A antibody on cells, Recombinant Human FGF-21 (#100-42; PeproTech; Rocky Hill, NJ, USA) was used at 100 ng/mL, lipopolysaccharide (LPS, from Escherichia coli, Sigma Aldrich, USA) at 1 µg/mL, (-)-parthenolide (#S2341, Selleckchem, TX, USA) at 100 ng/mL, and InVivoMAb anti-mouse IL-17A (#BE0173, clone 17F3, Bio X Cell, Lebanon) was used at 100 ng/mL to treat cell for 24 h. An assay was performed to co-culture indirectly the 3T3-L1 cells with the benign and malignant hepatic cell lines (Hepa1-6 cells, FL83B cells, Hepa1-6-21KD cells and FL83B-21KD cells) using a Transwell plate. In brief, 3T3-L1 cells were differentiated up to 8 days, and then seeded at 1 × 10^5^ in the insert of Trans-well plate for 24 h. After treatment with lipopolysaccharide LPS (Sigma Aldrich, USA) at 1 µg/mL for 12 h, the 3T3-L1 cells were co-cultured with hepatic cells for additional 24 h.

### Human HCC tumor samples

The human HCC tissue samples were prospectively collected from 33 patients who had undergone HCC nodule resection along with corresponding adjacent benign tissues between 2002 and 2014 from the James Graham Brown Cancer Center Bio-Repository at the University of Louisville. The human HCC serum samples pre-operation and post-operation were collected from 8 patients at the hospital of the University of Louisville. A microscope examination of the cellular composition of hepatic tissue confirmed the diagnoses of HCC and benign on these liver tissues reviewed by two pathologists independently, blinded to the subject's clinical history. All the human sample collection procedures for this study were approved by the Institutional Review Board for Human Study at the University of Louisville.

### GEPIA Database Analysis

Gene Expression Profiling Interactive Analysis (GEPIA), an online tool, was used to provide key interactions and functions based on the Cancer Genome Atlas (TCGA) and Genotype-Tissue Expression (GTEx) dataset for transcriptomic analysis (http://gepia.cancer-pku.cn) [PMID: 28407145]. In the GTEx dataset, the gene expression higher than median was defined as high expression group while the gene expression lower than median was defined as low expression group. By using the GEPIA tool, survival analyses were performed according to the gene expression levels of FGF21 and IL-17A using a log-rank test for hypothesis evaluation. GEPIA was further performed to determine a pairwise gene correlation analysis from any given set of TCGA and/or GTEx expression data using Pearson correlation statistics. The threshold was determined according to the following values: *P*-value of 0.01 and the hazard ration (HR) with 95% confidence intervals. Log-rank *P*-values were also calculated. The Spearman method was used to determine the correlation coefficient.

### Statistical analysis

Collected data from repeated experiments were presented as mean ± SD. Statistical analysis was performed by using SPSS V.17.0. Statistical significance was determined by ANOVA. The post hoc Tukey's test was used for analysis of any differences between groups. Group difference was considered significant for* P* < 0.05 (*),* P* < 0.01(**).

## Supplementary Material

Supplementary figures and tables.
