# Supplementary material for: Lack of FGF21 promotes NASH-HCC transition via hepatocyte-TLR4-IL-17A signaling
Source: Theranostics. 2020 Aug 7;10(22):9923–36. doi: 10.7150/thno.45988 (PMC7481424; doi:10.7150/thno.45988)
Supplement: Supplementary file 1 — Supplementary figures and tables. [file thnov10p9923s1.pdf]

**Supplementary materials**

Table 1 *List of antibodies*

| Antibody | Source (catalog) |
|---|---|
| IL-17A | Abcam (#ab79056) |
| TLR4 | Cell Signaling Technology (#14358S) |
| NF-κB | Cell Signaling Technology (#8242S) |
| IL-6 | Cell Signaling Technology (#12912S) |
| IL-23 | Invitrogen (#PA5-20239) |
| STAT3 | Cell Signaling Technology (#9139S) |
| RORγt | Invitrogen (#PA5-23148) |
| FASN | Abcam (#ab22759) |
| PPARα | Abcam (#ab3484) |
| FGF21 | Abcam (#ab171941) |
| IL-17RA | Invitrogen (#PA5-34571) |
| YAP/TAZ | Cell Signaling Technology |
| phospho-YAP Ser127 | Cell Signaling Technology |
| TGF-β | Cell Signaling Technology |
| Lipocalin2(LCN2) | Abcam (#ab63929) |
| IRS1 | Abcam (#ab52167) |
| 8-OHdG | Abcam (#ab48508) |
| F4/80 | Abcam (#ab16911) |
| γH2A.X s139 | Abcam (#ab2893) |
| Anti-mouse IgG | Cell Signaling (#7076S) |
| Anti-rabbit IgG | Cell Signaling (#7074S) |
| GAPDH | Santa Cruz (#FL-335) |

Table 2 Primers of the target genes

| Name | Gene ID | Sequence (5‘-3’) mouse | | Product length |
|---|---|---|---|---|
| TNF-α | 21926 | Forward | 5'-CTCATGCACCACCATCAAGGACTC-3' | 160 |
| | | Reverse | 5'-TCTGGAAAGGTCTGAAGGTAGGAAGG-3' | |
| IL-1β | 16176 | Forward | 5'-TTCAGGCAGGCAGTATCACTCATTG-3' | 169 |
| | | Reverse | 5'-ACACCAGCAGGTTATCATCATCATCC-3' | |
| IL-6 | 16193 | Forward | 5'-GAGGATACCACTCCCAACAGACC-3' | 141 |
| | | Reverse | 5'-AAGTGCATCATCGTTGTTCATACA-3' | |
| IL-23a | 83430 | Forward | 5'-GCACCTGCTTGACTCTGACATCTT-3' | 198 |
| | | Reverse | 5'-CGAAGGATCTTGGAACGGAGAAGG-3' | |
| IL-17A | 16171 | Forward | 5'-TGATGCTGTTGCTGCTGCTGAG-3' | 189 |

| | | | | |
|---|---|---|---|---|
| | | Reverse | 5'-TGGAACGGTTGAGGTAGTCTGAGG-3' | |
| TLR4 | 21898 | Forward | 5'-GGAGAATCCTGTGGACAAGGTCAG-3' | 143 |
| | | Reverse | 5'-ACACTCAGACTCGGCACTTAGCA-3' | |
| STAT3 | 20848 | Forward | 5'-GGAAAAGGACATCAGTGGCAAG-3' | 289 |
| | | Reverse | 5'-GGTATTGCTGCAGGTCGTTG-3' | |
| RORγt | 19885 | Forward | 5'-CCTTCACCCAGCCTTTCCCTTTCT-3' | 122 |
| | | Reverse | 5'-CCATCACTTGCTGCTGTTGTCCTAC-3' | |
| ASCL1 | 14081 | Forward | 5'-TTTTCTGATTCTGCTGCGGTG-3' | 298 |
| | | Reverse | 5'-CCATCAGTGGTACCCGCTATTT -3' | |
| Slc27a1 | 26457 | Forward | 5'-ATGTGCTCTATGACTGCC-3' | 164 |
| | | Reverse | 5'-TATGTACTGCACTACCGTG-3' | |
| Slc27a2 | 26458 | Forward | 5'-GAGGATACAAGATACCATTGAG-3' | 194 |
| | | Reverse | 5'-GGAATATTCAGAGGTTCAGAG-3' | |
| Slc27a3 | 26568 | Forward | 5'-AATCTGAAACCTTCCACTTG-3' | 86 |
| | | Reverse | 5'-ACAAAAGATACCCGAAAACC-3' | |
| Slc27a4 | 26569 | Forward | 5'-CTCAGCTATCTGTGAGATCC-3' | 172 |
| | | Reverse | 5'-GAGCTTATCTGTAAAACCCTTG-3' | |
| Slc27a5 | 26459 | Forward | 5'-ACCTCTGTACCATACGATAG-3' | 146 |
| | | Reverse | 5'-CCACATACAAGATCACTGTTAC-3' | |
| CD36 | 12491 | Forward | 5'-CATTTGCAGGTCTATCTACG-3' | 182 |
| | | Reverse | 5'-CAATGTCTAGCACACCATAAG-3' | |
| Acads | 11409 | Forward | 5'-CGTAGAGCTCTCGGTGTTCG-3' | 123 |
| | | Reverse | 5'-GACCAACTCCTTCTCGGCAA-3' | |
| Acadm | 11364 | Forward | 5'-AAAAGAGCCTGGGAACTCGG-3' | 169 |
| | | Reverse | 5'-GAATCACAGGCATTTGCCCC-3' | |
| PPARα | 19013 | Forward | 5'-GATGTCACACAATGCAATTC-3' | 107 |
| | | Reverse | 5'-CAGTTTCCGAATCTTTCAGG-3' | |
| Acox1 | 11430 | Forward | 5'-CATGTGGTTTAAAAACTCTGTGC-3' | 123 |
| | | Reverse | 5'-GGCATGAAGAAACGCTCCTG-3' | |
| Cpt1a | 12894 | Forward | 5'-TGCCTCTATGTGGTGTCCAA-3' | 300 |
| | | Reverse | 5'-CATGGCTTGTCTCAAGTGCT-3' | |
| Mttp | 17777 | Forward | 5'-TGCAAAATAGCGGTCACACA-3' | 125 |
| | | Reverse | 5'-TTTGTAGCCCACGCTGTCTT-3' | |
| Apoa1 | 11806 | Forward | 5'-GCACGTATGGCAGCAAGATG-3' | 149 |
| | | Reverse | 5'-GATTCAGGTTCAGCTGTTGGC-3 | |
| Dgat1 | 13350 | Forward | 5'-TAGAAGAGGACGAGGTGCGA-3' | 237 |
| | | Reverse | 5'-TCAGGATCAGCATCACCACAC-3' | |
| Acat1 | 110446 | Forward | 5'-CTGGGCGCAGGTTTACCTAT-3' | 182 |
| | | Reverse | 5'-GGTGTTGCTCCTCTGCTCAT-3' | |
| FASN | 14104 | Forward | 5'-TTGGCCTACACCCAGAGCTA-3' | 245 |

| | | | | |
|---|---|---|---|---|
| | | Reverse | 5'-TTGTGGTAGAAGGACACGGC-3' | |
| ACC1 | 107476 | Forward | 5'-ATGCGATCTATCCGTCGGTG-3' | 224 |
| | | Reverse | 5'-CCAGCCCACACTGCTTGTA-3' | |
| ACC2 | 100705 | Forward | 5'-TTCCCCAGCCAGCAGATAGC-3' | 147 |
| | | Reverse | 5'-CTTCATGTAGCCACGGGTCC-3' | |
| SREBP1 | 20787 | Forward | 5'-CAGCAGGTCCCAGTTGTACT-3' | 174 |
| | | Reverse | 5'-GATGGTCCCTCCACTCACCA-3' | |
| PPARγ | 19016 | Forward | 5'-AAAGACAACGGACAAATCAC-3' | 195 |
| | | Reverse | 5'-GGGATATTTTTGGCATACTCTG-3' | |
| GAPDH | 14433 | Forward | 5'-GTGAAGGTCGGTGTGAACGGATT-3' | 151 |
| | | Reverse | 5'-CGTGAGTGGAGTCATACTGGAACAT-3' | |

Table 3. Information of TCGA Patients

| | | | |
|---|---|---|---|
| **Gender** | | **Age** | |
| Male | 250 | ≥55 | 249 |
| Female | 119 | <55 | 120 |
| **BMI** | | **TNM** | |
| ≥24 | 209 | T1 | 180 |
| <24 | 160 | T2 | 95 |
| **Stage** | | T3 | 81 |
| I | 185 | T4 | 13 |
| II | 93 | N0 | 251 |
| III A | 66 | NX | 118 |
| III B | 10 | M0 | 310 |
| III C | 10 | MX | 59 |
| IV | 5 | | |

Survival curve data from TCGA were obtained from Gene Expression Profiling Interactive Analysis (GEPIA) [Tang Z, Li C, Kang B, Gao G, Li C, Zhang Z. GEPIA: a web server for cancer and normal gene expression profiling and interactive analyses. Nucleic Acids Res. 2017;45:W98–W102. doi: 10.1093/nar/gkx247.], which is based on the University of California Santa Cruz (UCSC) Xena project (http://xena.ucsc.edu).

## Supplementary Methods

Oil-Red-O staining

Oil Red O staining for lipid accumulation in cells was performed. In brief, after treatment, the cells in 24-well plate were washed with PBS and fixed in 4% buffered formalin for 5 min at room temperature. The cells were stained with Oil Red O dye for 1 h. All the images were reviewed and analyzed under microscope at 20x magnification.

ELISA assay

The protein levels of FGF21 and IL-17A were determined using an ELISA assay kit (R&D Systems,

DY3057, Inc. Minneapolis, MN) according to the manufacturer's instructions. In brief, a 96-well plate was coated with 100 µL per well of the diluted Capture Antibody (4 µg/mL) and incubated overnight at room temperature. Next day, 100 µL dilution of protein standards (15.6-1000 pg/mL) and samples were added into the wells and incubated for 2 h at room temperature. After washing, 100 µL of Detection Antibody (25 ng/mL) was applied for 2 h at room temperature and then 100 µL of the working dilution of Streptavidin-HRP (1: 200) was added to each well and incubated for 20 min at room temperature. Substrate Solution was added for 20 min at room temperature, and stopped by Stop Solution. Optical density (OD) was determined using a microplate reader at 450 nm and 540 nm/570 nm for correction. Concentration of the protein was calculated based on standard curve.

<u>Isolation of cells from the tissues of liver and spleen</u>

For hepatocyte and Kupffer cell isolation, liver was perfused at 5 mL per min with PBS containing EGTA (2.5 mM) and then digested with PBS containing Collagen D (3 mg/mL, #121-728-040; Roche), NaCl (66.7 mM), 4-(2-hydroxyethyl)-1-piperazineethanesulfonic acid (HEPES; 50 mM), and $CaCl_2$ (4.8 mM). The digested liver was dissected and then gently teased with forceps to suspend the cells in the solution. Cell suspension was filtered through 100-µm nylon cell strainer (BD Biosciences, Franklin Lakes, NJ) and centrifuged at 50 g speed for 2 min, and repeated for 3 times to collect hepatocytes for culture. The top aqueous phase was transferred into a new centrifuge tube and filtered through 70-µm nylon cell strainer (BD Biosciences, Franklin Lakes, NJ). The suspension was centrifuged with Percoll™ PLUS (GE Healthcare, Sweden #17-0891-01) at 1350 g for 10 min at 4 °C to collect non-hepatic parenchymal cells including Kupffer cells, sinusoidal endothelial cells, and satellite cells. The collected cells were washed with DMEM supplemented with 10% FBS, and then were seeded into rat tail collagen (Sigma, USA) precoated, 100-mm tissue-culture plates for 30 min, nonadherent cells were removed by aspiration, and fresh medium was added for Kupffer cell culture.

For the splenocyte isolation, in brief, a piece of spleen tissue was placed directly into the DMEM in a petri dish and homogenized between the frosted ends of two sterilized slides. The homogenized spleen was then transferred through a 70 µm cell strainer and mounted on a 50 mL tube. The cell strainer was rinsed with 5-10 mL DMEM to collect the cells. The suspended cells were then centrifuged at 800 g for 3 min. The red blood cells were removed by Ammonium-Chloride-Potassium lysis buffer.

<u>Establishment of FGF21 knockdown cell lines</u>

A mouse liver cell line, FL83B (ATCC CRL-2390), and a mouse hepatoma cell line, Hepa1-6 (ATCC CRL-1830), were used for the in vitro study. To establish FGF21 gene knock down (FGF21KD) cell lines, fourth generation lentivirus packing system (Lenti-X, Takara-Clontech) was used to generate FGF21 gene knock down (FGF21KD) cell lines of Hepa1-6 and FL83B. Briefly, puromycin-resistant shFGF21 (Sigma-Aldrich, MO) vectors were obtained, amplified, and purified for packing. For generating lentivirus, the lenti-X reagent was mixed with 6 µg of shRNA plasmid and transfected in 293T cell line for packing. The harvested lentivirus was used to transduce the cells using polybrene with established protocol. The cells were transduced with shRNA targeting FGF21 (shFGF21) (NM_020013/TRCN0000067373, Sigma-Aldrich), or sham sequences (shControl) (SHC002, Sigma-Aldrich) according to manufacturer's instructions. The Hepa1-6-21KD and the Hepa1-6-shControl (shCT) cells were cultured in DMEM (Gibco, USA), while the FL83B-21KD and FL83B-shCT cells were cultured in F12K medium (ATCC® 30-2004™). The FGF21KD cells were selected by puromycin (Sigma-Aldrich) and expanded after confirmation of gene knockdown by qRT-PCR and Western blot analysis.

<u>Flow Cytometry</u>

Flow Cytometry assay was performed in the single-cell suspensions of isolated cells from liver and spleen. In brief, cytokine stimulation was performed using 2 uL/mL eBioscienceTM Cell

Stimulation Cocktail (plus protein transport inhibitors) (#00-4975, Invitrogen, USA) for 16 h before cell staining. The cells were then stained with monoclonal antibodies or isotype controls. In brief, the cells were stained with APC-conjugated anti-mouse CD4 (#553051, eBiosciences, USA) for 20 min at 4 °C, followed by incubation with fixation buffer (#00-8222-49, eBiosciences, USA) and permeabilization buffer (#00-8333-56, eBiosciences, USA) according to the manufacturer's instructions. After twice washing with PBS, the cells were stained with FITC-conjugated anti-mouse IL-17 (#A15377, Life technologies molecular probes, USA) or PE-conjugated anti-mouse Foxp3 (#1946535, eBioscience, USA) for 1 h at room temperature. The cells were resuspended in PBS buffer to run flow cytometry. Flow cytometry data were then collected using a FACSCalibur (BD Pharmingen) and analyzed using FlowJo X software (vX0.7, Tree Star, San Carlos, CA) to evaluate the percentages of Th17 cells and Treg cells.

RNA extraction and real-time–polymerase chain reaction (RT-PCR)

Total RNA was extracted using the TRIzol reagent (Invitrogen, CA). First-strand complimentary DNA (cDNA) was synthesized from total RNA according to the kit protocol provided by manufacturer (Promega, Madison, WI). Quantitative PCR was carried out using the ABI 7300 real-time PCR system (Applied Biosystems, Carlsbad, CA). The target mRNA expression was quantified, and β-actin was used as an endogenous reference. For the list of primers, see Table 2 in the supplemental file. The $2^{-\Delta\Delta Ct}$ method was used to determine gene quantification with β-actin used as an endogenous reference gene. Results were expressed as fold change in gene expression compared to the WT-CD mice.

Western blot analysis

The protein levels were semi-quantified by Western blot analysis. In brief, electrophoresis was performed on 12% SDS-PAGE gel and the proteins were transformed to nitrocellulose membrane. The membranes were incubated with the primary antibodies (see Table 1 antibody list in supplemental file) overnight at 4℃ and with secondary antibody for 1 h at room temperature. The antigen-antibody complex was then visualized using ECL kit (Amersham, Piscataway, NJ). The protein bands were quantified by densitometry analysis and protein expression was presented pixel ratio of target protein vs endogenous reference, GAPDH or β-actin.

Immunohistochemistry staining analysis

Immunohistochemical staining was carried out on the paraffin-embedded material using the DAKO EnVision+System Kit. In brief, the sections were deparaffinized and hydrated. The slides were washed with a TRIS-buffer, and peroxidase blocking was performed for 5 min. After rewashing, the primary antibodies were applied for 60 min and then incubated with labeled polymer for 30 min at room temperature. The substrate-chromogen solution (diaminobenzidine) was added as a visualization reagent. Digital images were acquired with the Olympus 1×51 microscope (Olympus, Pittsburgh, PA) at 20x magnification using the Olympus DP72 digital camera and the length of scratch-wound was measured via the cellSens Dimention imaging system. The procedure for the computer image analysis was performed, and the acquired color images from the immunohistochemical staining were defined a standard threshold according to the software specification. The computer program then quantified the threshold area represented by color images. Protein expressions were defined by the percentages of threshold area in acquired color.

3T3-L1 differentiation

3T3-L1 cell line was purchased from ATCC (#CL-173). 3T3-L1 cells were cultured in DMEM medium containing 10% FBS. When cell growth reached to 80% confluence, the medium was replaced with the differentiation induction medium (1 mM dexamethasone, 0.5 mM Isobutyl-1-methylxanthine, 10 μg/mL insulin, DMI) for 2 days, then cells were cultured in maintenance medium (DMEM supplemented with 10 μg/mL insulin). The cell culture medium was changed

every 2 days up to 8 days. After differentiation, the cells were cultured in transwells (Corning Biocoat, #354570) which were inserted into transwell plates for co-culture with hepatic cells.

## Supplementary Figures

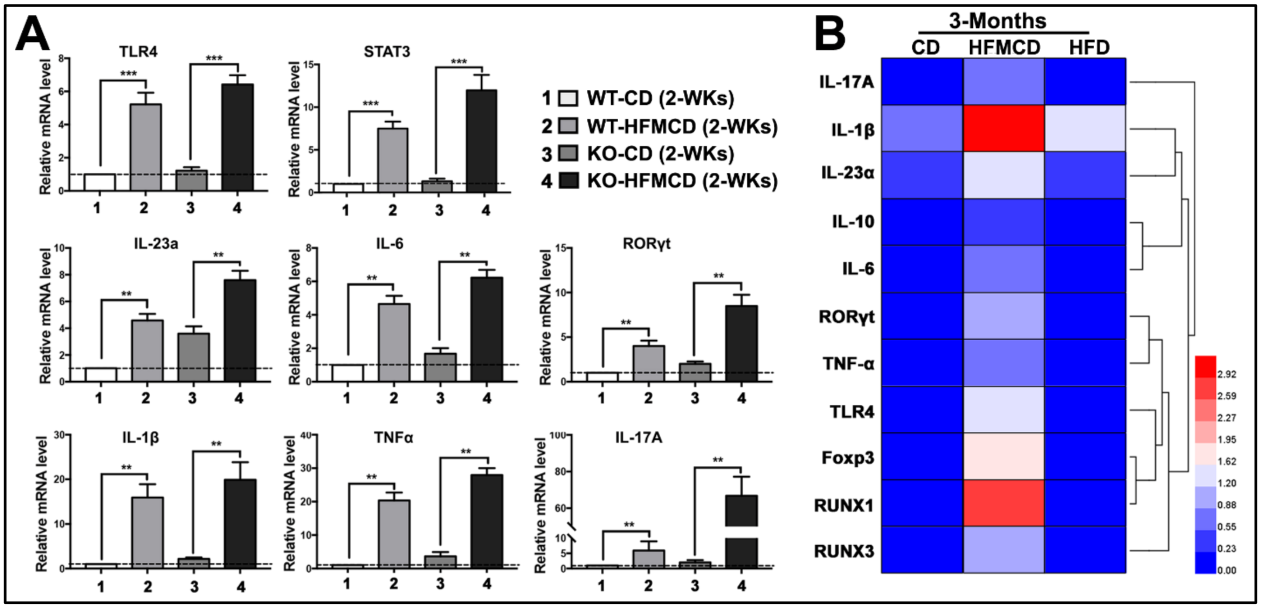


**Figure S1: A:** Relative mRNA levels of TLR4, STAT3, IL-23a, IL-6, RORγt, IL-1β, TNF-α andIL-17 were analyzed by qPCR in liver tissues from 4 groups (WT-CD; WT-HFMCD; FGF21KO-CD; FGF21KO-HFMCD. 2-weeks). Quantification for the mRNA levels is represented as the fold changes of β-actin. **B:** Heat map for the relative mRNA levels of IL-17A, IL-1β, IL-23a, IL-10, IL-6, RORγt, TNF-α, TLR4, Foxp3, RUNX1 and RUNX3 were analyzed by qPCR in liver tissues from FGF21KO mice with 3-moths feeding (CD, HFMCD and HFD). **, $P < 0.01$; ***, $P < 0.001$.

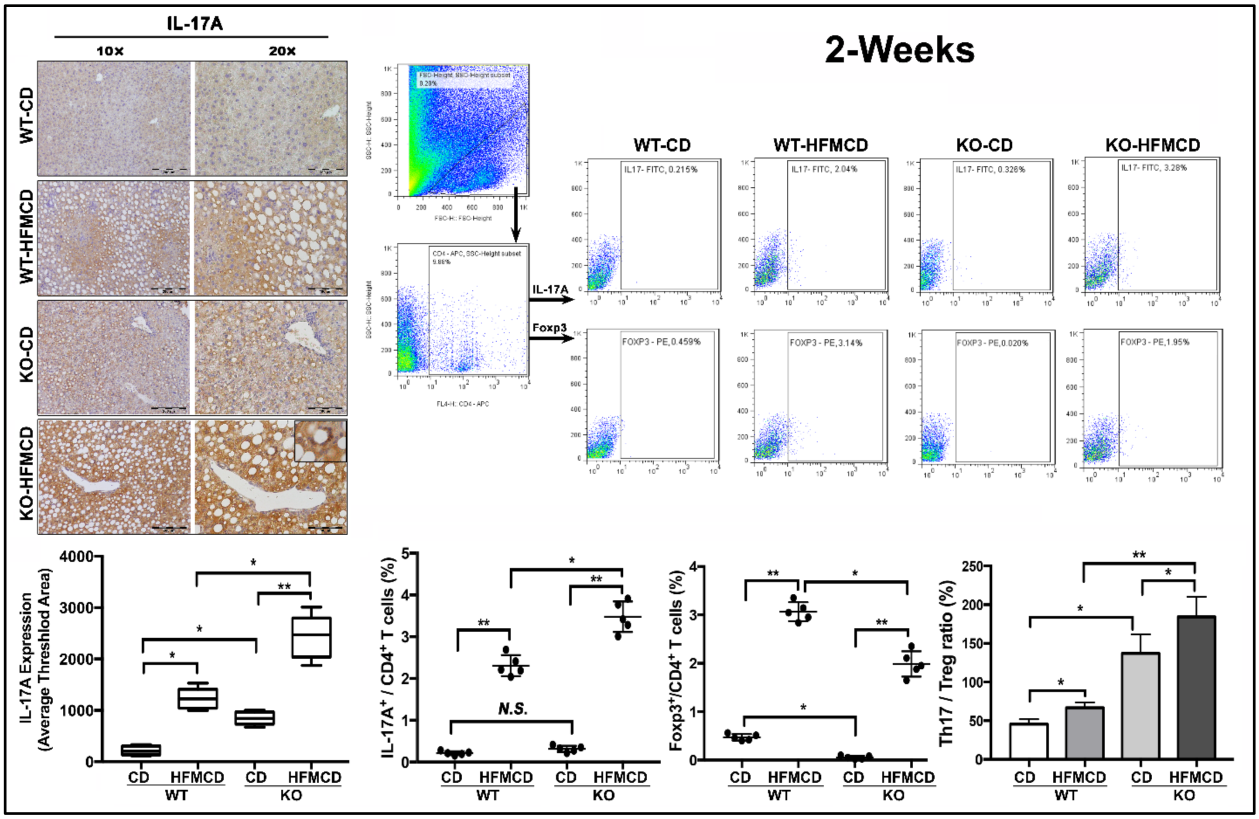


**Figure S2. Left:** Representative images of IL17A expression by immunohistochemical staining and computer image-quantification for the protein levels of IL17A in liver tissues from 4 groups (WT-CD; WT-HFMCD; FGF21KO-CD; FGF21KO-HFMCD. 2-weeks). **Right:** The Th17 population represented as $CD4^+$/$IL\text{-}17^+$ cells and the Treg population represented as $CD4^+$/$Foxp3^+$ cells were evaluated by flow cytometry in the isolated cells from liver tissues of 4 groups. The ratio of Th17 population over Treg population was determined. *N.S.*: no statistical significance; KO: FGF21KO; *, $P < 0.05$; **, $P < 0.01$.

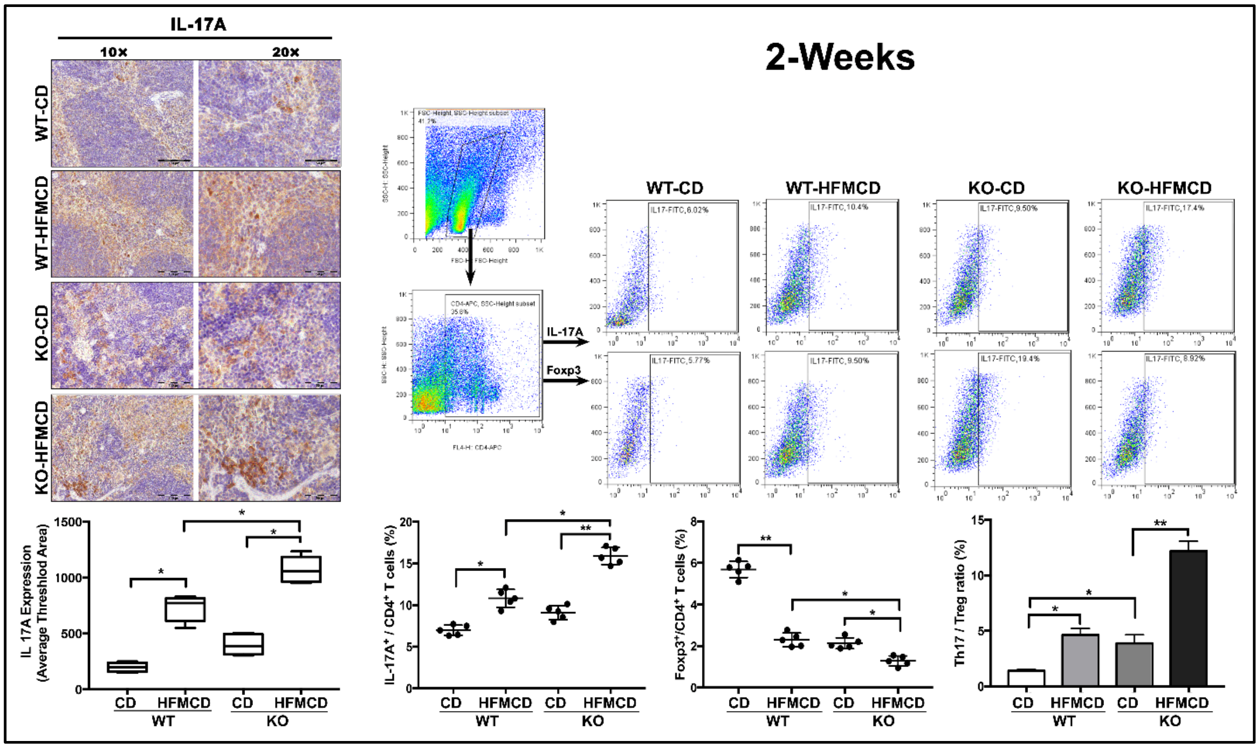


**Figure S3. Left:** Representative images of IL17A expression by immunohistochemical staining and computer image-quantification for the protein levels of IL17A in spleen tissues from 4 groups (WT-CD; WT-HFMCD; FGF21KO-CD; FGF21KO-HFMCD. 2-weeks). **Right:** The Th17 population represented as CD4$^+$/IL-17$^+$ cells and the Treg population represented as CD4$^+$/Foxp3$^+$ cells were evaluated by flow cytometry in the isolated cells from spleen tissues of 4 groups. The ratio of Th17 population over Treg population was determined. KO: FGF21KO; *, *P* < 0.05; **, *P* < 0.01.

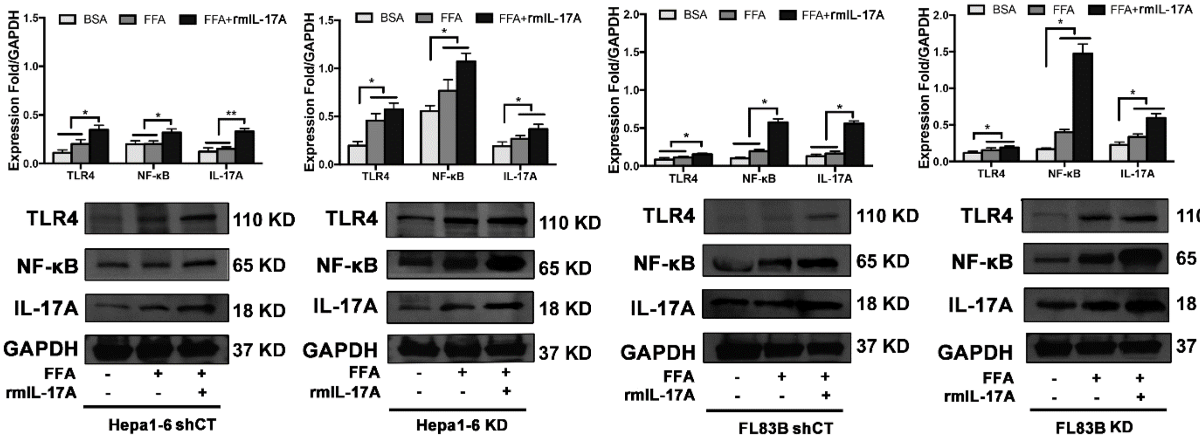


**Figure S4:** Western blot analysis for the protein levels of TLR4, NF-κB and IL-17A in the FL83B-FGF21KD cells and Hepal-6-FGF21KD cells treated with FFA and rmIL-17A. shCT: shRNA control; KD: FGF21 Knock down; FFA: free fatty acid; rmIL-17A: recombinant mouse IL-17A; *, *P* < 0.05; **, *P* < 0.01.

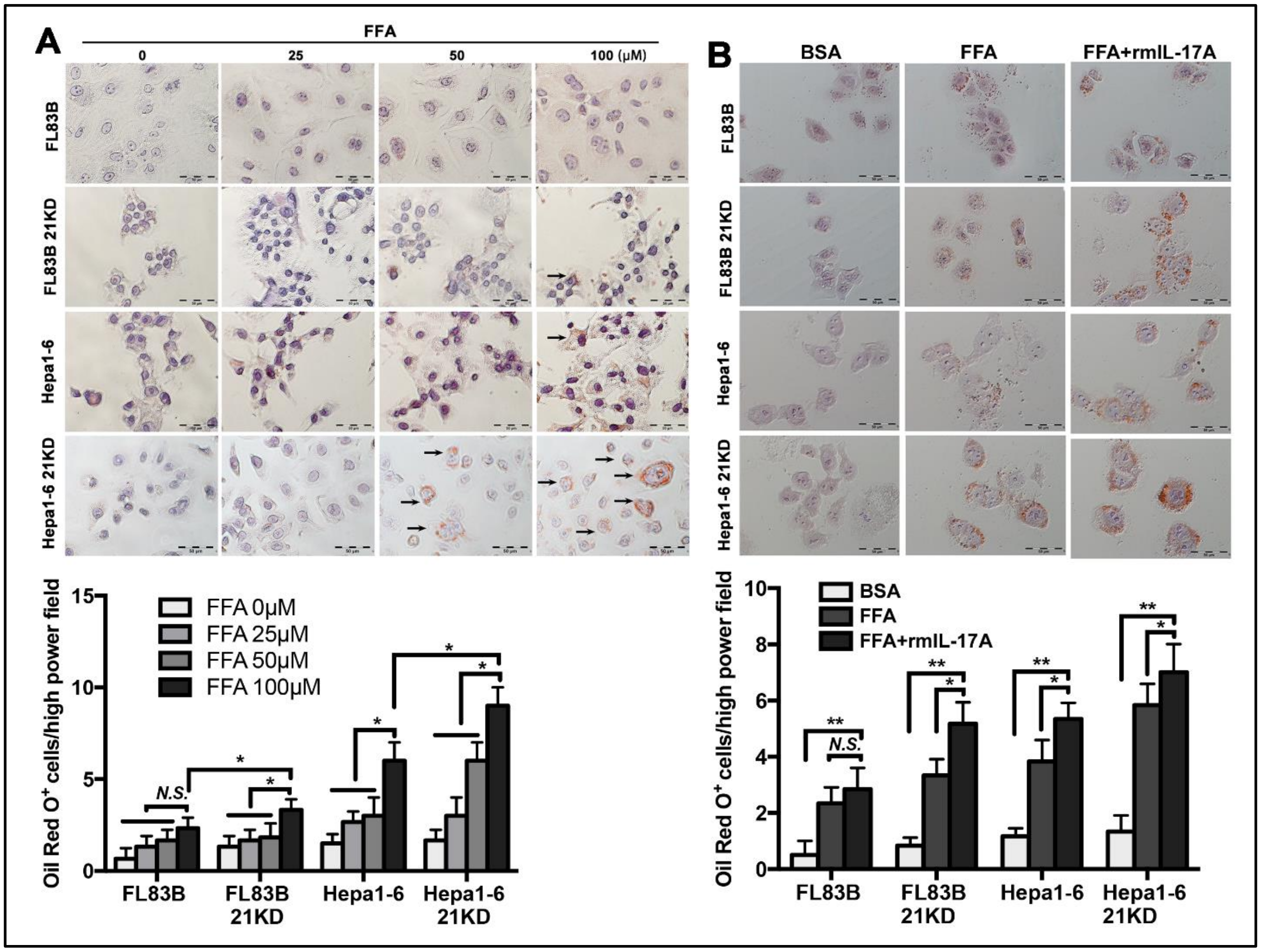


**Figure S5: A:** FL83B cells, FL83B-FGF21KD cells, Hepal-6 cells and Hepal-6-FGF21KD cells challenged with FFA (palmic acid) at 0, 25, 50, and 100 uM for 48 h. After washing, Oil Red O staining was performed to detect lipid accumulation in the cells. Positive Oil Red O staining cell was counted in high power field (40X), and the indexes of Red O staining positive cells were calculated. **B:** FL83B cells, FL83B-FGF21KD cells, Hepal-6 cells and Hepal-6-FGF21KD cells challenged with FFA (palmic acid) at 100 uM for 48 h, IL-17A was treated at 100 ng/mL, 1% BSA was used as treatment control. Positive Oil Red O staining cell was counted in high power field (40X), and the indexes of Red O staining cells were calculated. FFA: free fatty acid; 21KD: FGF21 knock down; BSA: bovine serum albumin; rmIL-17A: recombinant mouse IL-17A; *N.S.*: no statistical significance; *, $P < 0.05$; **, $P < 0.01$.

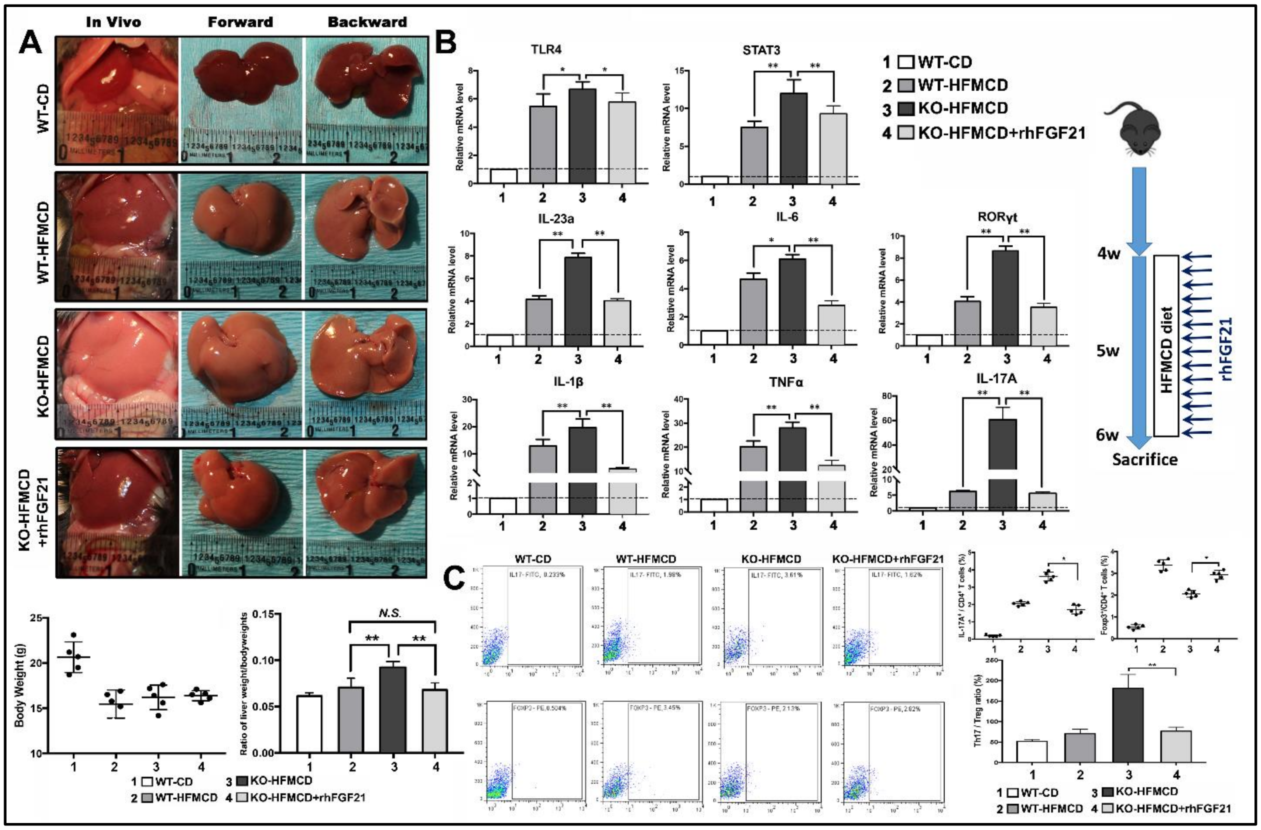


**Figure S6: A:** Representative gross anatomy of liver lobes, body weights and ratio of liver weight/bodyweights in the liver tissues from 4 groups (WT-CD; WT-HFMCD; FGF21KO-HFMCD; FGF21KO-HFMCD+rhFGF21). **B:** Relative mRNA levels of TLR4, STAT3, Il23a, IL-6, RORγt, IL-1β, TNF-α andIL-17 were analyzed by qPCR, represented as the fold changes of β-actin. **C:** The Th17 population represented as $CD4^+$/$IL\text{-}17^+$ cells and the Treg population represented as $CD4^+$/$Foxp3^+$ cells were evaluated by flow cytometry in the isolated cells from liver tissues of 4 groups (WT-CD; WT-HFMCD; FGF21KO-HFMCD; FGF21KO-HFMCD+rhFGF21). The ratio of Th17 population over Treg population was determined. *N.S.*: no statistical significance; KO: FGF21KO; *, $P < 0.05$; **, $P < 0.01$.

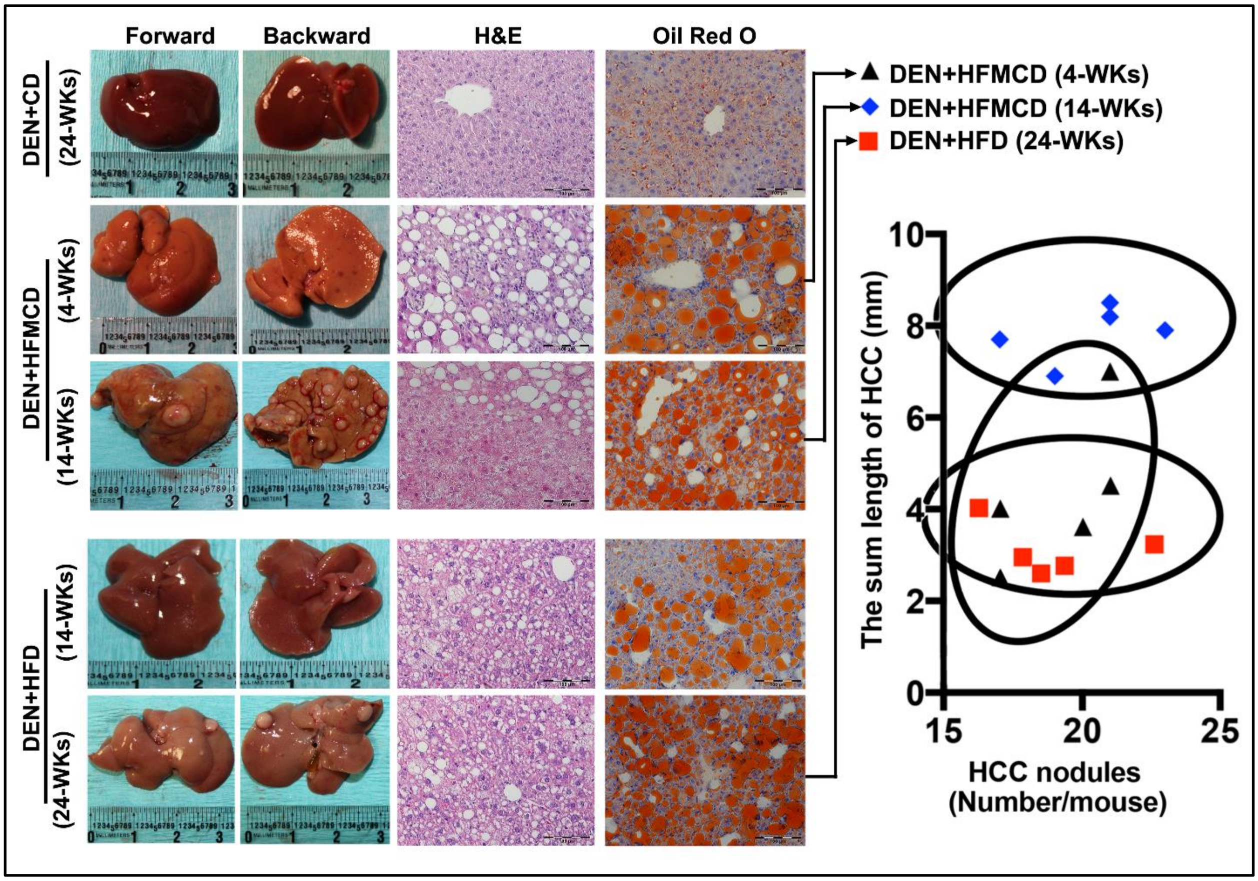


**Figure S7.** Representative gross anatomy of HCC nodules and histology by staining of H&E Oil Red O from 2 NASH-HCC models with EDN+HFMCD and DEN+HFD treatments. NASH-HCC tumor growth pattern represented as the regression of HCC nodule length and HCC nodule numbers in individual HCC mouse. HCC nodule appeared at 4 weeks and multiple HCC nodules were detected at 14 weeks in EDN+HFMCD mice, while scattered HCC nodules were detected at 24 weeks in DEN+HFD mice.
